# Spatiotemporal nigrostriatal iron accumulation in motor subtypes of Parkinson’s disease: from early to late stage

**DOI:** 10.1093/braincomms/fcaf473

**Published:** 2025-12-03

**Authors:** Shuting Bu, Huize Pang, Xiaolu Li, Yu Liu, Mengwan Zhao, Juzhou Wang, Lina Huang, Qin Niu, Le Liang, Hongmei Yu, Guoguang Fan

**Affiliations:** Department of Radiology, the First Hospital of China Medical University, Shenyang 110001 Liaoning, P.R. China; Department of Radiology, the First Hospital of China Medical University, Shenyang 110001 Liaoning, P.R. China; Department of Radiology, the First Hospital of China Medical University, Shenyang 110001 Liaoning, P.R. China; Department of Radiology, the First Hospital of China Medical University, Shenyang 110001 Liaoning, P.R. China; Department of Radiology, the First Hospital of China Medical University, Shenyang 110001 Liaoning, P.R. China; Department of Radiology, the First Hospital of China Medical University, Shenyang 110001 Liaoning, P.R. China; Department of Radiology, the First Hospital of China Medical University, Shenyang 110001 Liaoning, P.R. China; Department of Radiology, the First Hospital of China Medical University, Shenyang 110001 Liaoning, P.R. China; Department of Radiology, the First Hospital of China Medical University, Shenyang 110001 Liaoning, P.R. China; Department of Neurology, the First Hospital of China Medical University, Shenyang 110001 Liaoning, P.R. China; Department of Radiology, the First Hospital of China Medical University, Shenyang 110001 Liaoning, P.R. China

**Keywords:** iron, nigrostriatal system, quantitative susceptibility mapping, Parkinson’s disease, motor subtypes

## Abstract

The spatiotemporal ordering of nigrostriatal iron deposition across different motor subtypes of Parkinson’s disease (PD) remains poorly understand. This study explored the time course of nigrostriatal iron accumulation in 55 patients with postural instability and gait difficulty (PIGD) subtype and 53 patients with tremor-dominant (TD) subtype at early to late disease duration and 47 age-matched healthy controls (HC). Using quantitative susceptibility mapping, iron content was assessed in the substantia nigra (SN) and striatum. A spatial function method was employed to map the iron gradient along the principal axis of the subcortical structure. Nigrostriatal iron was compared among HC, PIGD/TD subgroup defined by disease duration [early, (<2 years); middle (2–6 years); late (>6 years)]. Associations with iron levels and motor symptoms were explored using partial correlation analysis. Nigrostriatal iron followed an inverted U-shaped progression, increasing initially and decreasing later in PIGD subtype. An S-shaped pattern was observed in TD subtype, increasing initially, decreasing later, then increasing again as disease progression. Iron distribution evolution of PD motor subtypes demonstrated opposite trends along medial-lateral axis (M-L) of the SN and anterior-posterior (A-P) axis of the putamen. Correlation analysis revealed that nigra iron content was positively associated with motor impairments, while caudate and putaminal iron level were negatively correlated with PIGD scores and Hoehn and Yahr scale in PIGD and TD subtype, respectively. These findings suggest that distinct nigrostriatal iron spatiotemporal pattern underlying different pathophysiology mechanism involved in these two PD motor subtypes. The opposite iron evolution trends along M-L axis of the SN and A-P axis of the putamen may provide a target for the development of new preventive or disease-modifying therapies.

## Teaser

Bu *et al*. reported distinct spatiotemporal iron accumulation patterns in Parkinson’s disease motor subtypes: an inverted U-shape in postural instability and gait difficulty subtype and an S-shape in tremor-dominant subtype as disease progressed, with opposite spatial shifts along medial-lateral axis of substantia nigra and anterior-posterior axis of putamen.

## Graphical Abstract

**Graphical Abstract:**
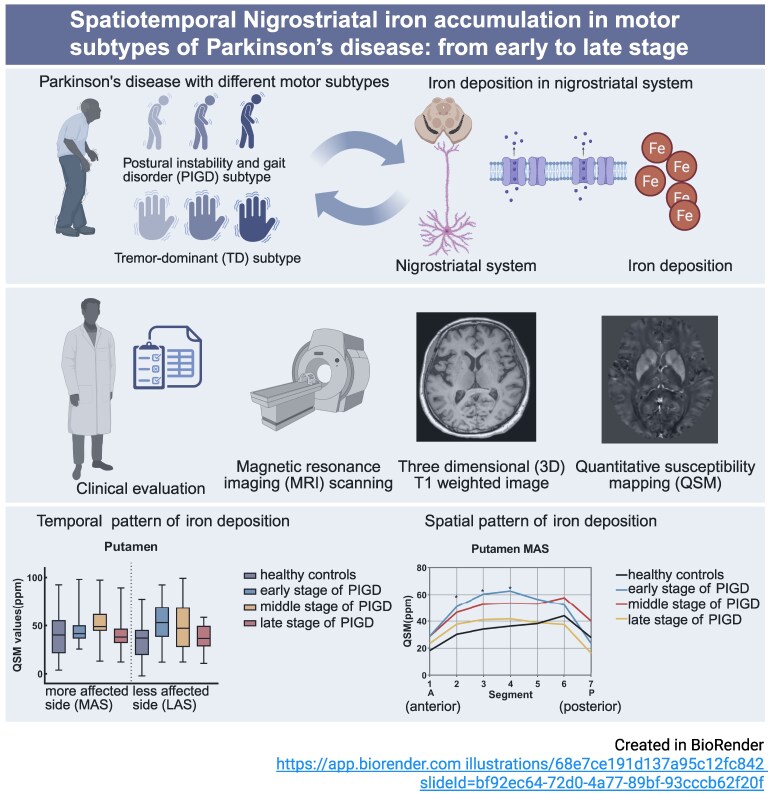

## Introduction

Parkinson’s disease (PD) is a neurological disorder characterized by progressive loss of dopaminergic neuromelanin-containing cells in the substantia nigra pars compacta (SNc) and diminished striatal dopaminergic function associated with increased iron deposition in the substantia nigra (SN).^1,2^ According to the Unified Parkinson’s Disease Rating Scale (UPDRS), PD is clinically classified into tremor-dominant (TD), postural instability and gait difficulty (PIGD), or mixed (MIX) subtypes.^3^ Among these, the PIGD patients is linked to a faster disease progression, increased risk of complications, more severe motor impairments, and less responsiveness to levodopa compared to those with TD subtype.^4^ Despite these distinctions, the spatiotemporal pattern of iron deposition within the nigrostriatal system between these two groups and how it affects PD motor symptoms remains obscure.

Iron plays a crucial role in various physiological processes, including the synthesis and metabolism of dopamine.^2^ However, excessive iron in its free radical form may exert neurotoxic effects by promoting the generation of reactive oxygen species, leading to oxidative stress, DNA damage, and ultimately trigger neuronal death.^5,6^ In PD, elevated iron levels within the SN have been confirmed using a range of techniques, including histochemistry,^7^ transcranial sonography^8^ and MRI,^9,10^ supporting its potential as a biomarker for neurodegeneration.^10-12^ Many studies have assessed iron content at the level of entire nigrostriatal nuclei and reported correlations between iron accumulation in regions such as the SN and putamen with motor symptom severity in PD.^13-16^ However, findings regarding differences in iron deposition between PD motor subtypes remain inconsistent. Some studies have identified higher iron content in the substantia nigra pars reticulata (SNr) and SNc in patients with PIGD subtype compared to those with TD subtype,^14^ while others found no significant differences in SNr iron levels between these two groups.^17^ These discrepancies may stem from methodological limitations, such as reliance on manual segmentation and the assumption of homogeneity within nuclei, which can obscure region-specific variations in iron distribution. Moreover, the progression of iron accumulation is known to be nonlinear and stage-dependent,^18^ and its distribution within the nigrostriatal system is spatially heterogeneous.^19,20^ Consequently, it remains unclear whether iron accumulation precedes or follows the onset of PD and how it evolves across disease stages in different motor subtypes. Clarifying the spatiotemporal dynamics of iron deposition in the nigrostriatal system may offer valuable insights into the distinct neuropathological mechanisms underlying PD motor subtypes.

Quantitative susceptibility mapping (QSM) is an advanced MRI technique that allows for precise quantification of tissue magnetic susceptibility, and is now considered the gold standard for in vivo assessment of brain iron content.^21^ Due to its superior sensitivity and accuracy compared to R2*, QSM has been extensively employed to investigate iron accumulation in PD.^22^ Importantly, traditional analyses often overlook the spatial heterogeneity of iron distribution within subcortical nuclei. To overcome this limitation, a region of interest (ROI) principal axis-based spatial segmentation method utilizing a singular value decomposition (SVD) algorithm has been developed.^23^ This method redefines the three-dimensional coordinates of each nucleus based on its morphological structure and enables equidistant spatial segmentation. A recent study successfully applied this technique to characterize nigrostriatal iron gradients in various age-related synucleinopathies.^24^ Building upon this approach, the present study further investigates iron spatial progression patterns in the nigrostriatal system across distinct PD motor subtypes.

Here, we employed a SVD algorithm to measure the spatiotemporal distribution of iron content within the bilateral nigrostriatal system across different motor subtypes of PD, from early to late stage. We hypothesized that (1) the temporal ordering of nigrostriatal iron concentrations in the PIGD would differ with TD subtype as disease progressed; (2) spatial distribution of iron accumulation along three main axes within the nigrostriatal system differs between PD motor subtypes; (3) nigrostriatal iron deposition would be significantly correlated with specific clinical motor symptoms.

## Materials and methods

**Figure 1:**
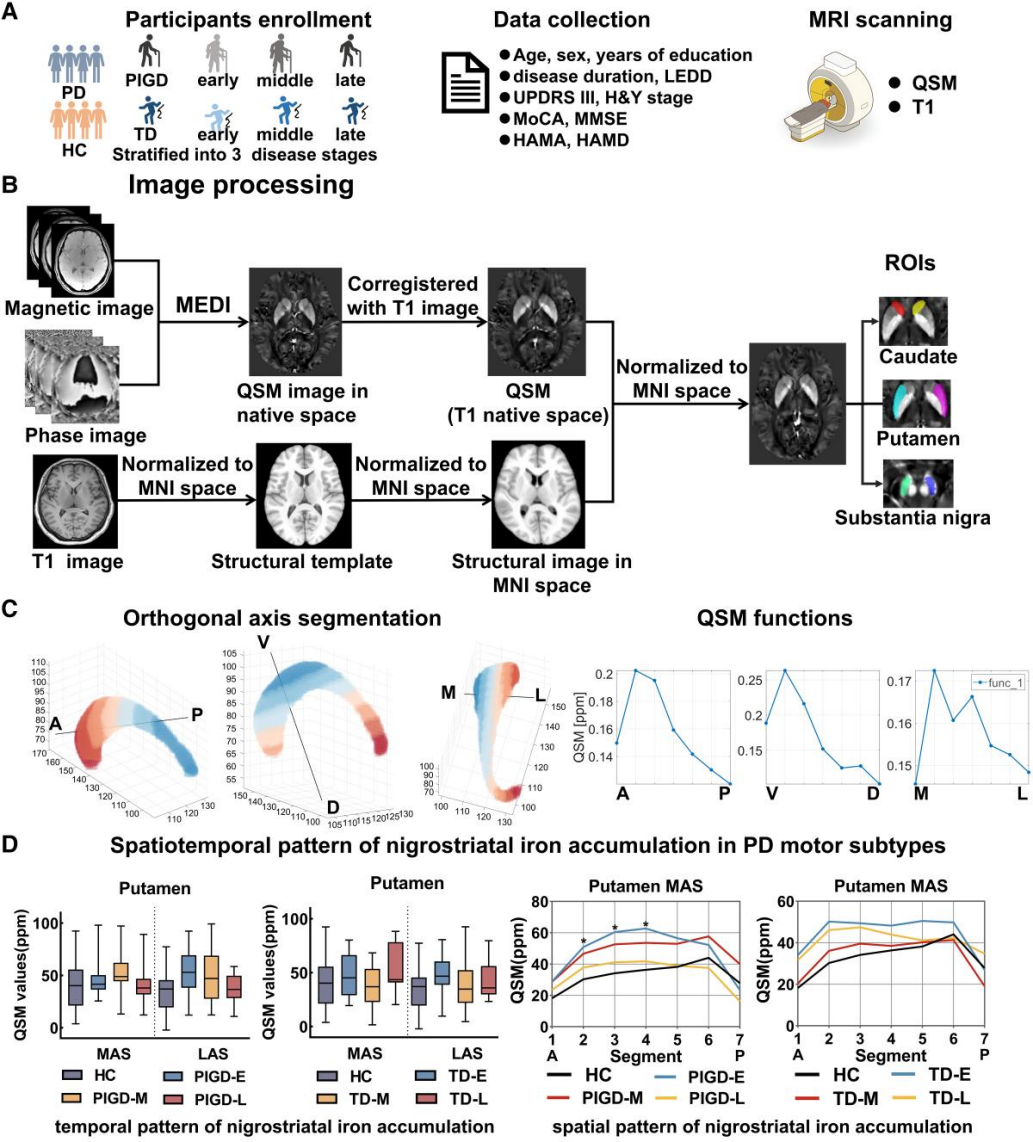
**Flowchart for the design of this study**. (**A)** participants enrollment, clinical data collection and MRI scanning. (**B)** QSM images preprocessing and ROI selection. (**C)** The ROI axis-based spatial segmentation approach (https://github.com/MezerLab/mrGrad) was used to segment ROIs equidistant along three orthogonal axes, and QSM functions were finally generated. (**D)** temporal and spatial pattern of nigrostriatal iron accumulation in PD motor subtypes. A, Anterior; ANTs, Advanced Normalization Tools; D, Dorsal; L, Lateral; MNI, Montreal Neurological Institute; MEDI, Morphology Enabled Dipole Inversion; M, Medial; PD, Parkinson’s disease; PIGD, postural instability and gait difficulty subtype; PIGD-E, early disease stage of PIGD; PIGD-M, middle disease stage of PIGD; PIGD-L, late disease stage of PIGD; P, Posterior; QSM, Quantitative susceptibility mapping; ROIs, Regions of interest; TD, tremor-dominant subtype; TD-E, early disease stage of TD; TD-M, middle disease stage of TD; TD-L, late disease stage of TD; V, Ventral.

All participants provided informed consent, and the study was approved by the Institutional Review Board of China Medical University. The flowchart of this study was shown in Fig. 1.

### Participants

Patients meeting the UK PD Society Brain Bank Criteria^25^ were recruited to the study from the Department of Neurology at the First hospital of China Medical University between January 2023 to March 2025. Motor subtype classification was based on the ratio of the mean tremor score (11 items) to the mean PIGD score (5 items) derived from the UPDRS. Patients with a ratio ≥1.15 were classified as TD subtype, those with a ratio ≤0.9 as PIGD subtype, and those with a ratio >0.9 and <1.15 as intermediate subtype.^26^ Demographic information from each participant including age, sex, years of education, and Levodopa Equivalent Daily Dose (LEDD) were collected prior to MRI scanning. All PD patients underwent comprehensive clinical assessments. Movement Disorder Society-sponsored revision of the UPDRS (MDS-UPDRS) Parts III, as well as the Hoehn and Yahr (H&Y) tests were performed for motor symptoms assessment. Cognitive function was evaluated using the Mini-Mental State Examination (MMSE) and Montreal Cognitive Assessment (MoCA) tests. Besides, depression and anxiety symptoms were assessed with the Hamilton Depression Rating Scale (HAMD) and the Hamilton Anxiety Scale (HAMA), respectively. Meanwhile, age- and sex-matched healthy controls (HC) were recruited from the local community. Inclusion criteria for all participants were: (i) 40 to 55 years old; (ii) Han ethnicity; (iii) right-handedness. Exclusion criteria included a history of other neurological or psychiatric disorders, classification as an intermediate subtype, MRI contraindications, and abnormal findings on MRI. Preliminary, 47 HC (17 males, 30 females), 60 PIGD patients (28 males and 32 females), 58 TD patients (34 males, 24 females) were recruited in our study. In PD patients, the body side of first motor symptoms was defined as the clinically most affected.

### Subgroup definition

PD duration was defined based on the time since a neurologist first diagnosed the disease. To investigate the progression of nigrostriatal iron accumulation over the course of PD, both PIGD and TD patients were stratified into three disease stages: (i) Early-stage, defined as disease duration of <2 years; (ii) Middle-stage, defined as a duration of 2 to 6 years; representing the so-called ‘honeymoon’ phase during which patients often remain clinically ‘stable’.^27,28^ (iii) Late-stage, defined as a disease duration >6 years; corresponding to the post- ‘honeymoon period’ characterized by more pronounced clinical decline.

### MRI acquisition

A 3.0 Tesla scanner (MAGNETOM Vida; Siemens Healthineers, Erlangen, Germany) with a 64-channel head coil was used to perform the MRI scans. The MRI examination consisted of an axial three-dimensional gradient-echo (3D-GRE) sequence and a sagittal three-dimensional T1-weighted magnetization-prepared rapid gradient echo sequence with inversion recovery (MPRAGE) sequence. The scanning protocol was as follows: (i) 3D-MPRAGE: echo time (TE) = 1880 ms, repetition time (TR) = 1870 ms, flip angle = 9^◦^, inversion time = 940 ms, slice number = 176, slice thickness = 1.0 mm, field of view (FOV) = 256 mm × 256 mm, voxel size = 1 × 1 × 1 mm^3^; (ii) 3D-GRE: TR = 50 ms; TE = 6.15, 11.7, 17.25, 22.8, 28.35, 33.90, 39.45 and 45 ms; flip angle = 15°; FOV = 220 mm × 220 mm; voxel size = 0.9 × 0.9 × 2 mm^3^.

### QSM preprocessing and spatial standardization

QSM was reconstructed from phase and magnitude images acquired using a 3D-GRE sequence. Image processing was conducted using the fully automated Morphology Enabled Dipole Inversion (MEDI) MATLAB toolbox, with susceptibility values zero-referenced to the ventricular CSF. The default pipeline of the multi-scale dipole inversion algorithm was employed. Initially, the total magnetic field was estimated from the multi-echo data using a nonlinear fitting method.^29^ Subsequently, spatial field unwrapping and background field removal were achieved by projecting onto the dipole field algorithm, leading to the derivation of the local field.^30^ This local field was then inverted to compute the final susceptibility map. The numerical inversion process incorporated structural priors derived from the magnitude image (e.g. edges) and a regularization term to enforce uniform susceptibility within the CSF of the lateral ventricles to improve the quality of QSM and automatically standardize susceptibility relative to CSF.^31,32^

Skull stripping was performed on the 3D T1-weighted images and QSM maps using the Brain Extraction Tool from the Functional MRI of the Brain Software Library (FSL, version 10.0; http://www.fmrib.ox.ac.uk/fsl/). QSM images were then standardized to a common space using the Advanced Normalization Tools (ANTS, https://github.com/ANTsX/ANTs). The 3D T1-weighted images were first registered to the HCP-MMP (Human Connectome Project Multi-Modal Parcellation version 1.0) standard space using symmetric image normalization implemented in ANTS, generating the corresponding transformation matrices. To spatially normalize QSM images, co-registration between QSM images and the 3D T1-weighted images was performed. The resulting transformation matrices were subsequently applied to the QSM images, enabling their alignment to HCP-MMP. All QSM images were visually inspected to exclude those with poor quality or failed spatial normalization.

With all QSM images registered to the HCP-MMP, ROI for the bilateral SN, putamen, and caudate nucleus were defined based on the HCP-MMP for subsequent analyses. Mean QSM values were extracted from each bilateral ROI for each participant with fslmaths.

### QSM gradients mapping of subcortical Nuclei

#### Orthogonal axis calculation

Using the predefined ROI masks, the algorithm calculates the SVD of the voxel 3D image coordinates within the ROI to determine the principal three orthogonal axes of the structure.^23^ In the present study, three orthogonal axes of bilateral SN, putamen, and caudate nucleus were identified as the anterior-posterior (A-P), ventral-dorsal (V-D), and medial-lateral (M-L) axes. The SVD algorithm solution was described in detail in Supplementary material.

#### Orthogonal axis segmentation

Each ROI was partitioned into uniformly spaced segments along its three principal orthogonal axes. To achieve this, the boundaries of the data were defined by two hyperplanes orthogonal to the axis of interest, positioned at the outermost points along that axis. The region between these boundaries was then subdivided using *n*-1 equally spaced, parallel hyperplanes, resulting in *n* equidistant segments. Voxel coordinates within the ROI were assigned to one of the n segments based on their spatial location. In this study, we selected *n* = 7 for segmentation, consistent with prior studies that have applied this method to subdivide subcortical nuclei.^23^

#### QSM functions along the orthogonal axes of the ROI

For each QSM map, the median susceptibility value was calculated within each segment along every axis. This procedure yielded three ROI-based functions representing spatial positions along the three principal orthogonal axes of the designated ROI. These spatial functions were then averaged across subjects for each axis to generate group-averaged functions of spatial variations within each ROI.

### Statistical analysis

The Kolmogorov-Smirnov test (*n* > 50) was employed to assess the normality of data distributions. Continuous variables were expressed as means ± standard deviations (SD) and categorical variables as numbers (%). Demographic and clinical variables were compared among the HC, TD and PIGD groups. For normally distributed continuous variables, two-sample *t*-tests were used, whereas non-normally distributed quantitative variables were analyzed using the Mann–Whitney U test. Categorical variables were assessed using the chi-square test. Statistical analyses were performed using SPSS version 25.0 (IBM Software Analytics).

Mean ROI values were lateralized based on the predominance of motor symptoms in PD patients or hand dominance in HC, resulting in classification into more-affected and less-affected sides (MAS and LAS) for PD, and dominant and non-dominant sides (DS and nDS) for HC. General linear models (GLMs) were conducted for MAS (DS in HC) and LAS (nDS in HC) separately in order to asses group differences, with age, sex and LEDD as covariates. Initially, GLMs were applied to compare nigrostriatal iron deposition between HC and PD groups. Subsequently, PD participants were classified into PIGD and TD subtype to explore nigrostriatal iron levels among HC, PIGD and TD groups, with age, sex, and LEDD as covariates. Furthermore, GLMs were used to examine intergroup differences in iron gradients among HC, PIGD-E, PIGD-M, and PIGD-L, as well as among HC, TD-E, TD-M, and TD-L, again controlling for age, sex, and LEDD, to investigate spatiotemporal patterns of iron accumulation within each PD motor subtype. Bonferroni correction was applied to account for multiple comparisons and control the Type I error rate. Finally, associations between regional iron levels (mean QSM values) and clinical characteristics were assessed using partial correlation analyses within the SN, putamen and caudate with age as a covariate.

## Results

### Demographic and clinical characteristics

A total of 155 participants were included in the final analysis after excluding individuals due to head motion artifacts and incomplete clinical data. The cohort consisted of 47 HC and 108 PD patients (55 PIGD subtype and 53 TD subtype). Demographic and clinical characteristics of the three groups were presented in Table 1. There were no significant differences among PIGD, TD, and HC groups in terms of age, sex, or years of education. Additionally, no significant differences were observed between the TD and PIGD subtypes in total UPDRS III scores, MAS UPDRS III scores, LAS UPDRS III scores, disease duration, HAMA, or HAMD scores. However, MoCA scores were significantly lower in both PD subtypes compared to HC, with no differences between the TD and PIGD groups. Furthermore, the TD group demonstrated significantly lower LEDD and H&Y stage compared to the PIGD group. Details of medication status for all PD patients were presented in Supplementary Table 1.

**Table 1:** Demographic and clinical characteristics of the participants

Characteristics	HC (*n* = 47)	PIGD (*n* = 55)	TD (*n* = 53)	ANOVA	*Post hoc* test		
					HC versus PIGD	HC versus TD	PIGD versus TD
Age (y)	66.87 ± 7.11	64.44 ± 7.87	65.22 ± 6.05	0.222	0.087	0.268	0.583
Sex (M/F)	17/30	26/29	31/22	0.083	-	-	-
Education (y)	9.00 (9.00,12.00)	9.00 (9.00,12.00）	9.00 (7.50,11.50)	0.283	-	-	-
Side Of onset (R/L)	-	25/30	25/28	-	-	-	0.858
Disease duration	-	3.30 (2.00;7.00)	2.50 (1.63;5.63)	-	-	-	0.214
LEDD	-	400 (150.00;675.00)	250.00 (0.00;406.25)	-	-	-	**0**.**009^a^**
MoCA scores	26.00 (24.00,27.00)	24.00 (21.00;25.00)	22.00 (19.50,25.00)	**<0.001^b^**	**<0.001^b^**	**<0.001^b^**	0.987
MMSE scores	28.00 (27.00;290.00)	27.00 (25.00,29.00)	27.00 (24.00,29.00)	**0.034^a^**	0.208	**0.035^a^**	1.00
H&Y stage	-	2.00 (2.00;3.00)	2.00 (1.50;2.00)	-	-	-	**0**.**011^a^**
Tremor scores	-	3.00 (1.00,5.00)	10.00 (8.00,14.00)	-	-	-	**<** **0**.**001^b^**
PIGD scores	-	5.00 (3.00,8.00)	2.00 (1.00,3.00)	-	-	-	**<** **0**.**001^b^**
Total UPDRS III	-	36.00 (21.00,45.00)	33.00 (22.00,43.00)	-	-	-	0.495
MAS UPDRS III	-	16.00 (11.50,18.00)	14.00 (9.00,17.00)	-	-	-	0.070
LAS UPDRS III	-	9.00 (3.50,14.00)	9.00 (6.00,13.00)	-	-	-	0.983
HAMA scores	-	12.00 (7.50;17.00)	10.00 (6.00,15.75)	-	-	-	0.197
HAMD scores	-	12.00 (6.50,17.00)	10.00 (5.00,16.00)	-	-	-	0.196

HC, healthy controls; PIGD, postural instability and gait difficulty subtype; TD, tremor-dominant subtype. ANOVA, analysis of variance; M/F, male/female; LAS, less affected side; LEDD, Levodopa Equivalent Daily Dose; MAS, more affected side; MoCA, Montreal Cognitive Assessment; MMSE, Mini-Mental State Examination; H&Y, Hoehn and Yahr; UPDRS, Unified Parkinson's Disease Rating Scale; HAMA, Hamilton Anxiety Scale; HAMD, Hamilton Depression Rating Scale.

Note: Statistically significant *P* values are shown in bold.

^a^denotes *P* value less than 0.05.

^b^denotes *P* value less than 0.001.

### Iron content alterations in the nigrostriatal system of PD motor subtypes

In the comparison between PD and HC groups, PD patients showed higher QSM values than HC in the SN, caudate, putamen, MAS and LAS, respectively. However, these differences did not reach statistical significance after controlling for age, sex, and LEDD as covariates (Table 2, Supplementary Fig. 2). Similarly, when comparing HC, PIGD, and TD groups, the PIGD group exhibited the highest QSM values in the bilateral SN, caudate, and putamen, followed by the TD group, with the HC group showing the lowest values. Nonetheless, no statistically significant differences among the three groups were observed after adjusting for age, sex, and LEDD (Table 3, Supplementary Fig. 3).

**Table 2:** Comparisons of regional QSM values between HC and PD groups

Region	HC (*n* = 47)	PD (*n* = 108)	t	*P*
MAS-SN	95.63 ± 33.09	116.95 ± 40.62	−0.719	0.473
LAS-SN	87.81 ± 33.86	119.49 ± 41.9	−1.621	0.107
MAS-Putamen	39.17 ± 20.54	44.95 ± 18.92	−0.917	0.360
LAS-Putamen	34.88 ± 18.55	44.14 ± 20.67	−1.603	0.111
MAS-Caudate	28.06 ± 14.52	28.83 ± 14.45	−0.739	0.461
LAS-Caudate	27.09 ± 13.47	27.93 ± 14.11	−0.052	0.958

*Note*: HC, healthy controls; PD, Parkinson’s disease; LAS, less affected side; MAS, more affected side; SN, substantia nigra.

**Table 3:** Comparisons of regional QSM values among HC, PIGD and TD groups

Region	HC (*n* = 47)	PIGD (*n* = 55)	TD (*n* = 53)	F	*P*	*Post hoc* test		
						HC versus PIGD	HC versus TD	PIGD versus TD
MAS-SN	95.63 ± 33.09	118.24 ± 43.11	113.77 ± 41.27	0.545	0.581	0.332	0.708	0.440
LAS-SN	87.81 ± 33.86	122.72 ± 48.47	115.31 ± 36.80	1.682	0.190	0.069	0.256	0.355
MAS-Putamen	39.17 ± 20.54	46.72 ± 18.92	41.48 ± 20.13	2.953	0.055	0.255	0.972	0.231
LAS-Putamen	34.88 ± 18.55	47.09 ± 22.08	40.75 ± 20.44	3.031	0.051	0.231	0.609	0.255
MAS-Caudate	28.06 ± 14.52	29.80 ± 15.52	27.05 ± 14.35	0.877	0.418	0.255	0.785	0.269
LAS-Caudate	27.09 ± 13.47	29.18 ± 15.06	25.99 ± 14.09	0.850	0.430	0.531	0.674	0.195

*Note*: HC, healthy controls; PIGD, postural instability and gait difficulty subtype; TD, tremor-dominant subtype. LAS, less affected side; MAS, more affected side; SN, substantia nigra.

**Figure 2:**
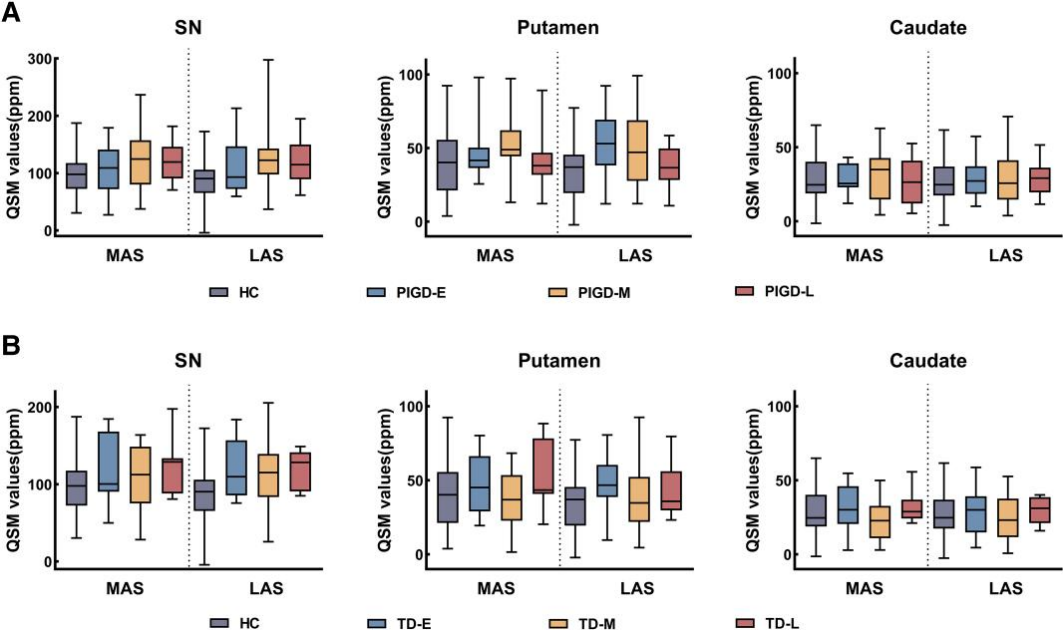
**Evolution of iron content in the nigrostriatal system. Bars indicate mean QSM values ± SD for each group**. (**A)** Boxplots illustrating QSM values for healthy controls (purple) and early, middle and late disease duration PIGD subjects (blue, yellow, and red, respectively) (N_HC_ = 47, N_PIGD-E_ = 11, N_PIGD-M_ = 28, N_PIGD-L_ = 16). Group differences were assessed by general linear model, adjusted for age, sex, and LEDD (for details, see Supplementary Table 3A). (**B)** Boxplots illustrating QSM values for healthy controls (purple) and early, middle and late disease duration TD subjects (blue, yellow, and red, respectively) (N_HC_ = 47, N_TD-E_ = 12, N_TD-M_ = 26, N_TD-L_ = 15). Group differences were assessed by general linear model, adjusted for age, sex, and LEDD (for details, see Supplementary Table 3B). LAS, less affected side, MAS, more affected side; PIGD, postural instability and gait difficulty subtype; PIGD-E, early disease stage of PIGD; PIGD-M, middle disease stage of PIGD; PIGD-L, late disease stage of PIGD; QSM, quantitative susceptibility mapping; SD, standard deviation; SN, substantia nigra; TD, tremor-dominant subtype; TD-E, early disease stage of TD; TD-M, middle disease stage of TD; TD-L, late disease stage of TD.

In PIGD patients, nigrostriatal iron progressively increased and then decreased along with disease stage in both hemispheres, MAS and LAS (Fig. 2A). In contrast, TD patients exhibited a fluctuating pattern, with iron accumulation increasing in the early stage, decreasing in the middle stage, and rising again in the late stage (Fig. 2B), although these differences across stages were not statistically significant in PIGD and TD group. Specifically, the SN consistently exhibited the highest iron content, followed by the putamen, while the caudate nucleus showed the lowest levels in both hemispheres. This pattern suggests a potential sequential progression of iron accumulation within the nigrostriatal system—beginning in the SN, extending to the putamen, and lastly affecting the caudate.

### Spatiotemporal distribution of iron accumulation in the nigrostriatal system of PD motor subtypes

To further investigate the spatiotemporal alterations of iron content within the nigrostriatal nuclei, we employed a SVD algorithm to redefine the three-dimensional coordinates and 7 segments of each nucleus. In the SN, iron deposition was primarily localized to the posterior segment along the A-P axis, the ventral segment along V-D axis, and the lateral segment along the M-L axis. In the putamen, iron accumulation was similarly concentrated in the posterior, ventral, and lateral regions. In contrast, iron deposition in the caudate nucleus was predominantly observed in the anterior segment along the A-P axis, the dorsal segment along the V-D axis, and the lateral segment along the M-L axis. These findings were similar to those we obtained in our studies of the distribution of nigrostriatal iron deposition in HC, PIGD and TD group (see Supplementary Fig. 1, Supplementary Table 2).

**Figure 3:**
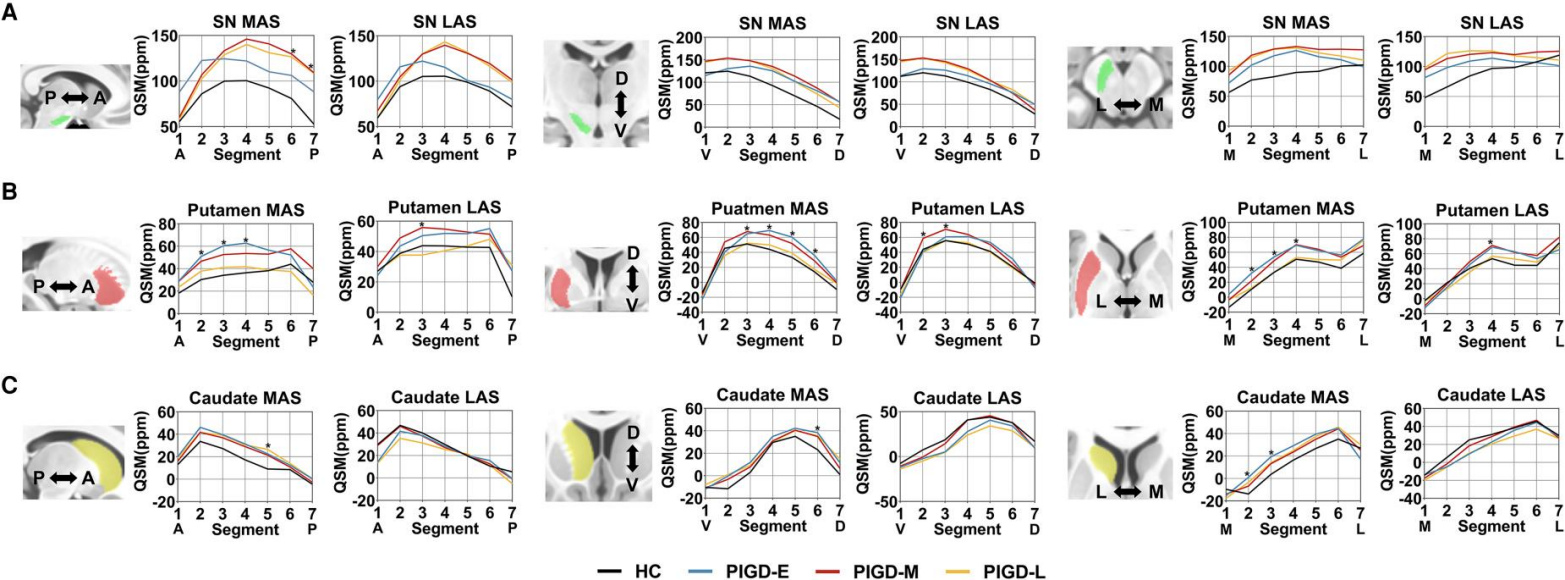
**Spatial iron deposition patterns in the nigrostriatal system across HC (black), and early, middle and late disease duration PIGD subjects (blue, red, and yellow, respectively). N_HC_ = 47, N_PIGD-E_ = 11, N_PIGD-M_ = 28, N_PIGD-L_ = 16. Lines show mean QSM values of all subjects at each segment within the group**. **(A)** gradients of SN iron deposition; **(B)** gradients of putamen iron deposition; **(C)** gradients of caudate iron deposition. Group differences were assessed with general linear models, adjusted for age, sex, LEDD (for details, see Supplementary Table 4). Segment represents the specific section along the axis. A, anterior; D, Dorsal; HC, healthy controls; LAS, less affected side; L, lateral; MAS, more affected side; M, medial; PIGD, postural instability and gait difficulty subtype; PIGD-E, early disease stage of PIGD; PIGD-M, middle disease stage of PIGD; PIGD-L, late disease stage of PIGD; P, posterior; SN, substantia nigra; V, Ventral. * denotes *p* value < 0.05 among four groups.

**Figure 4:**
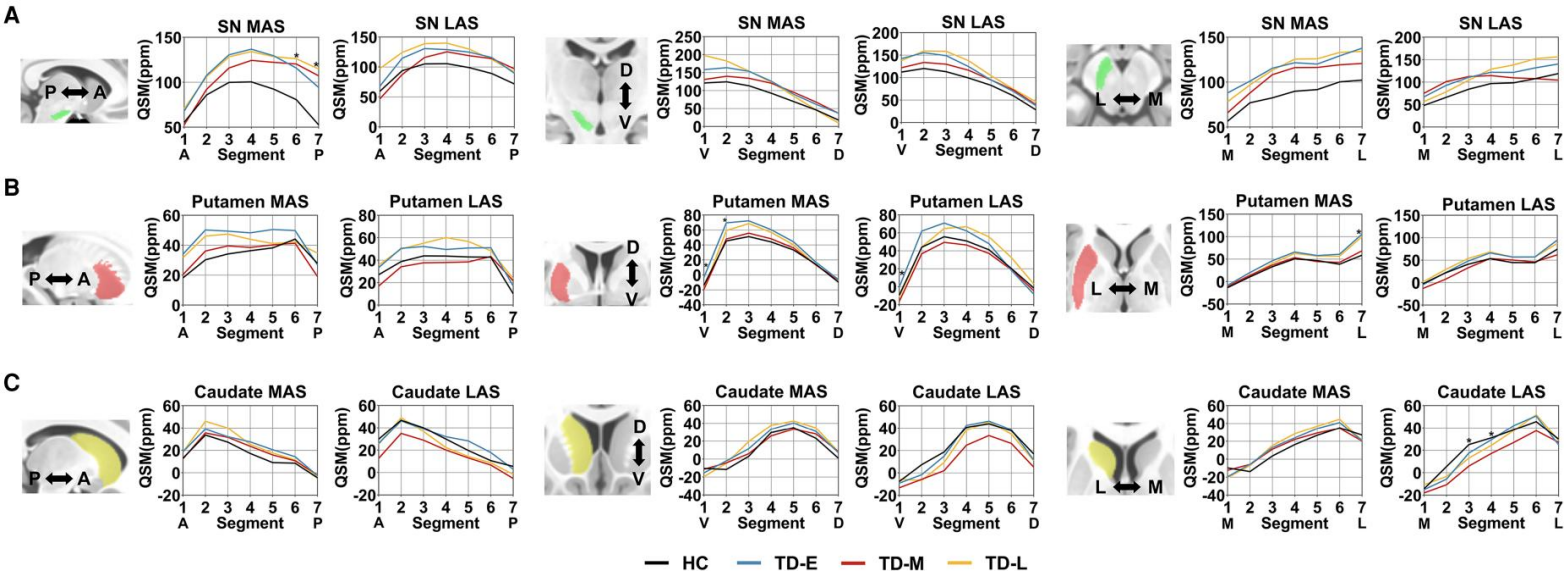
**Spatial iron deposition patterns in the nigrostriatal system across HC (black), and early, middle and late disease duration TD subjects (blue, red, and yellow, respectively). N_HC_ = 47, N_TD-E_ = 12, N_TD-M_ = 26, N_TD-L_ = 15. Lines show mean QSM values of all subjects at each segment within the group**. **(A)** gradients of SN iron deposition; **(B)** gradients of putamen iron deposition; **(C)** gradients of caudate iron deposition. Group differences were assessed with general linear models, adjusted for age, sex, LEDD (for details, see Supplementary Table 5). Segment represents the specific section along the axis. A, anterior; D, Dorsal; HC, healthy controls; LAS, less affected side; L, lateral; MAS, more affected side; M, medial; P, posterior; SN, substantia nigra; TD, tremor-dominant; TD-E, early disease stage of TD; TD-M, middle disease stage of TD; TD-L, late disease stage of TD; V, Ventral. * denotes *P* value < 0.05 among four groups.

As illustrated in Figs 3 and 4, on the MAS, the PIGD group exhibited a progressive shift in peak QSM values within the SN toward the posterior, ventral, and medial segments as the disease advanced. In contrast, the TD group demonstrated only a slight ventral shift in the late stage, with the peak moving from segment 2 to segment 1. On the LAS, both the PIGD and TD groups showed a posterior shift of peak QSM values along the A-P axis over time. However, along the M-L axis, the two groups showed divergent trajectories. In the PIGD group, the peak QSM values was located at segment 4 in the early stage, shifted laterally to segment 7 in the middle stage, and returned medially to segment 4 in the late stage. Conversely, the TD group showed an opposite pattern: the peak was initially located at the most lateral segment (segment 7), shifted medially to segment 4 in the middle stage, and reverted to the segment 7 in the late stage. These findings suggest opposing spatial dynamics of iron accumulation along the M-L axis between the two PD motor subtypes.

Regarding the peak QSM values in the putamen, both PIGD and TD subtypes demonstrated similar trajectories on the MAS, with posterior shifts from early to middle stages, followed by anterior shifts in the late stage along the A-P axis. On the LAS, the two subtypes diverged: the PIGD group showed a progressive posterior-anterior-to-posterior shift, while the TD group exhibited an anterior-posterior-to-anterior shift. Along the V-D axis, peak QSM values in both subtypes consistently localized to the ventral segment (segment 3). Along the M-L axis, lateral peak localization (segment 6) was observed in both groups on the MAS. However, on the LAS, the PIGD group showed a progressive lateral shift with disease progression, whereas the TD group displayed no substantial changes.

Notably, the spatial distribution of iron within the caudate nucleus remained consistent across both the MAS and LAS and showed no significant changes throughout disease progression.

Beyond evaluating the spatiotemporal patterns of iron deposition across motor subtypes, we also applied GLMs to assess intra-group spatial differences in iron distribution among HC, PIGD and TD patients at different disease stages, adjusting for age, sex, and LEDD as covariates (detailed information were showed in Supplementary Tables 4 and 5). In the SN, stage-related differences were predominantly localized to the posterior region on the MAS in both the PIGD and TD groups. In the PIGD group, putaminal differences were primarily observed in the anterior, dorsal, and medial segments of the MAS, and in the anteroventral region of the LAS. In contrast, the TD group exhibited spatial differences in the anterior and lateral segments of the putamen. For the caudate nucleus, the PIGD group showed variations in the posterior, dorsal, and medial segments on the MAS, whereas in the TD group, differences were restricted to the medial region of the LAS.

### Correlation between nigrostriatal iron deposition and clinical symptoms

**Figure 5:**
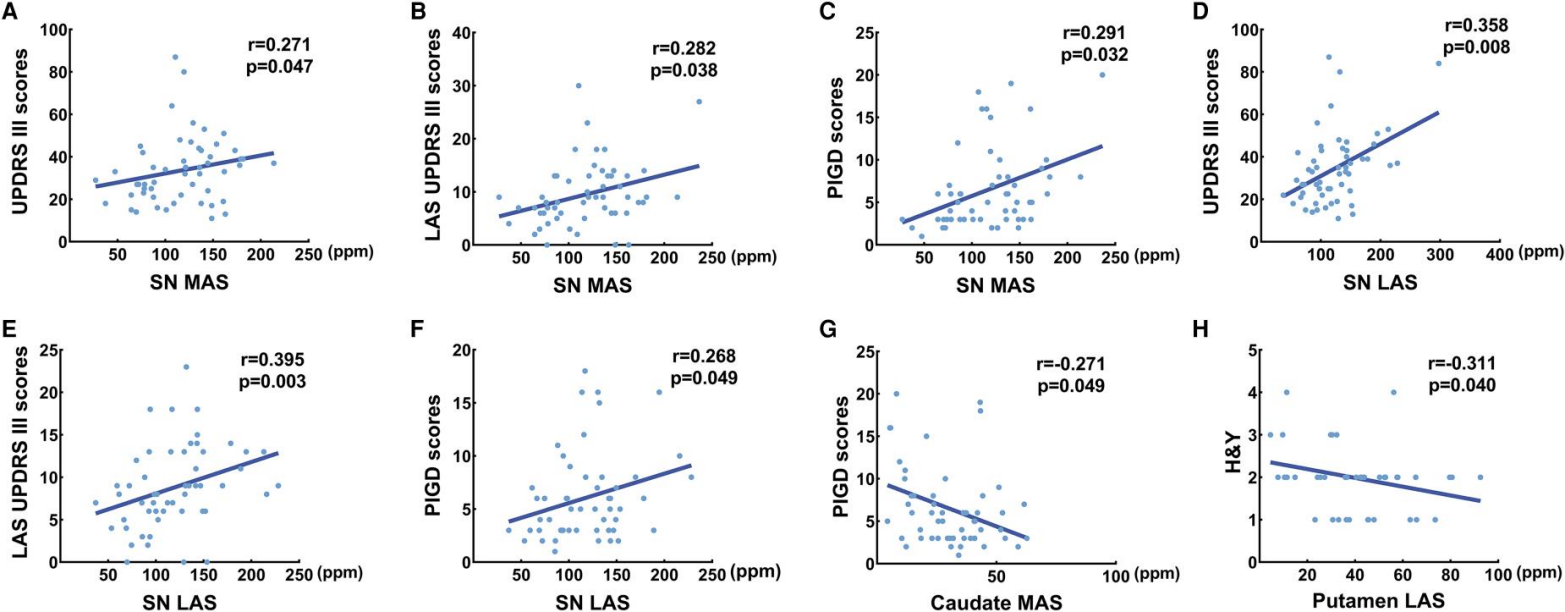
**Partial correlation analyses of nigrostriatal iron content and clinical characteristics in PIGD group** (**A–G)**, and TD group (**H**), adjusted for age. (N: PIGD = 55, TD = 53). Each point represents an individual participant, and the line indicates the best-fit linear regression. HC, healthy controls; H&Y, Hoehn and Yahr scale; LAS, less affected side; MAS, more affected side; PIGD, postural instability and gait difficulty; SN, substantia nigra; TD, tremor-dominant; UPDRS, Unified Parkinson’s Disease Rating Scale.

Partial correlation analyses were conducted to examine the associations between nigrostriatal iron deposition and clinical motor symptoms across PD motor subtypes with age as a covariate. In the PIGD subtype, QSM values in the SN showed significant positive correlations with PIGD scores, total UPDRS III scores, and LAS UPDRS III scores on both hemispheres (*P* < 0.05). Caudate iron content on the MAS was negatively correlated with PIGD scores in PIGD group (*P* = 0.049, r = −0.271), and putaminal iron level on the LAS was negatively associated with H&Y in TD group (*P* = 0.040, r = −0.311) (Fig. 5, FDR correction).

## Discussion

In this study, we investigated the progression of iron accumulation across different PD motor subtypes from early to late disease duration using QSM. Additionally, we further examined spatial distribution of iron within nigrostriatal nuclei with SVD methods. Our findings revealed distinct spatiotemporal patterns of iron deposition between the PIGD and TD subtypes. The key findings were as follows: (i) Iron accumulation in the nigrostriatal system followed an inverted U-shaped pattern as the disease progressed in the PIGD subtype, whereas an S-shaped pattern was observed in the TD subtype; (ii) Iron distribution along M-L axis of the SN and A-P axis of the putamen showed similar trajectories on the MAS while showed distinct trajectories on the LAS in the PIGD and TD subtypes; (iii) Iron content in the SN was positively correlated with motor symptoms in the PIGD subtype. While caudate iron content on the MAS and putaminal iron level on the LAS showed negatively associations with PIGD scores and H&Y in the PIGD group and TD group, respectively.

Nigral iron levels showed an initial increase in the early and middle stage, then decreased in the late stage in PIGD subtype, while it increased in the early stage, decreased in the middle stage, and a reaccumulation in the late stage in TD subtype, both MAS and LAS. The main contributor to iron increment in the nigra is probably neuronal death, as we observed in the early stage of two motor subtypes. Consistent with previous studies,^33,34^ we observed a decline in nigra iron levels during the late stage in the PIGD subtype, which may reflect a floor effect due to the extensive loss of nigra cells associated with disease progression.^33^ Besides, previous studies have shown that levodopa administration can effectively reduce iron accumulation in nigrosome 1 within the SN.^35^ Thus, as the disease progresses, the loss of nigra cells and the effects of antiparkinsonian medicine lead to a decline in iron deposition within the SN, beginning in the middle stage for the TD subtype and in the late stage for the PIGD subtype. Given that the TD subtype typically exhibits a better responsiveness to levodopa treatment compared with the PIGD subtype,^36,37^ the decline in nigral iron levels in TD may be more strongly influenced by levodopa than in PIGD subtype. Regarding the secondary increase in nigral iron observed in the late stage of the TD subtype, we hypothesize that this may be related to iron accumulation within tremor-associated circuits. The generation of tremor in PD has been associated with the cerebellar-thalamo-cortical circuit. Previous studies have demonstrated iron accumulation in key components of this circuit, including the red nucleus and dentate nucleus, particularly in PD patients with prominent tremor.^38^ Furthermore, an animal model study has shown that intravenous iron administration can induce parkinsonian-like tremor, with iron observed to cross the blood-brain barrier and accumulate in brain regions.^39^Notably, prior research demonstrated that plasma transferrin, the primary iron transport protein, was higher in TD subtype than non-TD individuals,^40^ and that iron burden correlated with tremor severity.^41^ Collectively, findings support a mechanism of late-stage iron reaccumulation in the TD subtype, possibly driven by sustained tremor circuit activity and transferrin-mediated transport. In summary, the inverted U-shaped pattern of nigral iron accumulation observed in the PIGD subtype may be primarily associated with progressive loss of nigra cells and the pharmacological influence of levodopa. In contrast, the S-shaped pattern of nigral iron accumulation in the TD subtype is likely the result of a combined effect of dopaminergic neuronal loss, the pharmacological influence of levodopa, and tremor-related circuit-specific iron redistribution.

We also observed that the striatum (putamen and caudate nucleus) in both PIGD and TD subtypes exhibited temporal patterns of iron deposition similar to those observed in the SN. In humans, iron concentrations increase with aging in the striatum,^42^ as we observed in this study. Experimental studies have demonstrated that direct injection of ferric iron into the SN reduces both dopamine content and release in the striatum,^43-45^ potentially explaining the similar patterns of iron accumulation between these regions. In addition, there is a correlation between iron accumulation and clinical motor symptoms, as evidenced by previous animal experiments.^46,47^ Similarly, our study demonstrated elevated iron levels in the SN were positively correlated with UPDRS III scores, LAS-UPDRS III scores, PIGD scores in the PIGD subtype, MAS and LAS, respectively. Besides, negative significant associations were detected between iron levels in the MAS-caudate and PIGD scores in the PIGD group, as well as between putaminal iron content on the LAS and H&Y in the TD group.

It is noteworthy that no significant differences in nigrostriatal iron content were observed across disease stages within either the PIGD or TD subgroups. Previous studies have reported heterogeneous findings regarding disease stage–related changes in nigrostriatal iron. For instance, Guan *et al*.^34^ observed a decrease in nigral iron deposition with disease progression, whereas Bergsland *et al*.^11^ demonstrated increased iron levels in the ventral posterior but not dorsal SN over a 3-year follow-up, or even no significant alterations of nigral iron was found longitudinally.^18,48^ Similarly, studies on striatal regions have yielded inconsistent results, ranging from no significant changes^9,10^ to marked increases or even decreases compared with HC.^11,34,49,50^ Several factors may explain the lack of significant group differences in our study: (i) The proportion of patients with advanced disease (disease duration >6 years) was relatively small. A postmortem study of 8 PD patients with an average disease duration of 7.5 years^51^ also failed to reveal a substantial increase in nigral iron deposition, suggesting that the patients in our cohort may not have exhibited sufficiently advanced nigral changes to yield detectable group-level differences. (ii) The overall sample size in our study was modest, and stratification by disease duration further reduced the number of participants per subgroup, which may have limited the statistical power to detect between-group differences. Currently, studies focusing on disease stage–related alterations in nigrostriatal iron deposition across different PD motor subtypes remains scarce. Despite the limited sample size and lack of longitudinal data, our findings offer preliminary insights into the temporal patterns of nigrostriatal iron deposition in different PD motor subtypes.

In addition to exploring the temporal pattern of iron deposition in the nigrostriatal system with disease progression, we also explored its spatial distribution characteristics. Across HC, and both PIGD and TD subtypes, including all disease stages, nigral iron was consistently concentrated in the posterior, ventral, and lateral segments. This finding aligns with neuropathological studies indicating that the posterior SN is affected earlier and more severely than the anterior SN in PD.^52^ We observed significantly elevated QSM values in the posterior SN of both PIGD and TD patients compared to HC, particularly in the middle and late stages, suggesting its potential utility as a biomarker for disease progression. Degeneration of dopaminergic neurons is greatest in the ventrolateral tier of the SNc and least in the dorsomedial tier.^53-55^ The ventrolateral tier, situated primarily lateral and posterolateral to the red nucleus,^55-57^ corresponds anatomically to the middle and posterior SN. High-resolution imaging studies using 7T MRI have demonstrated a gradient of increasing iron from the dorsal to the ventral SN,^58^ and increased iron concentration in the ventral SNc has also been reported.^59^ Unfortunately, we did not find any intergroup differences in ventral or lateral SN iron deposition, indicating the need for future investigations with larger sample sizes. Interestingly, we found that peak QSM values along the M-L axis of the SN exhibited divergent trajectories between PIGD and TD subtypes on the LAS as the disease progressed. Anatomically, the dorsolateral SN subregion is primarily connected with somatomotor and somatosensory cortices (pre-/post-central gyrus), while the dorsomedial SNc (medial SN) is predominantly linked to limbic areas such as the orbitofrontal cortex, hippocampus, and amygdala. Neuromelanin-sensitive imaging has shown greater signal loss in the lateral SN of PIGD patients compared to TD patients.^60^ Given that tremor is more closely associated with the cerebellar-thalamo-cortical circuit, whereas PIGD is more strongly linked to the nigrostriatal pathway, the opposite spatial trajectories of QSM peak values along the M-L axis likely reflect distinct pathophysiological mechanisms underlying these motor subtypes.

In addition, the putamen follows A-P iron deposition pattern. The lateral SN connects to the posterior putamen (motor striatum) via the posterior limb of the internal capsule. Correspondingly, we observed distinct trends in peak QSM values along A-P axis of putamen on the LAS. This aligns with evidence of inhomogeneous dopaminergic degeneration, which is most pronounced in the posterior putamen.^61-63^ Single-photon emission computed tomography (SPECT) studies^64-66^ have shown that patients with akinetic-rigid subtype exhibit lower dopamine uptake in the putamen compared to TD patients, with uptake levels correlating with the severity of rigidity and hypokinesia but not with tremor. These findings suggest that damage to the posterior putamen may underlie gait and postural impairments in PD. Unlike SN and putamen, peak QSM values in the caudate remained stable along A-P, V-D, M-L axes as disease progresses in both PIGD and TD subtype. Caudate iron deposition was primarily localized to the anterior, ventral, and lateral segments across HC, as well as PIGD and TD subtype, at all stage of disease progression. Prior QSM studies have reported inconsistent findings regarding caudate iron deposition in TD subtype.^13,17^ Several SPECT studies^67,68^ suggest that PD patients with tremor retain a relative higher caudate uptake than those without tremor, who often show near-normal caudate uptake.^67^ This relative preservation of dopaminergic neurons may explain the stable iron levels observed in the caudate as PD progresses.

Our study is not without its limitations. First, our sample size is not very large. Still, we applied rigorous inclusion criteria and conducted thorough quality checks on the QSM images and image registration processes to minimize confounding factors that could affect the measurement of iron gradients. We believe these steps enhance the reliability and robustness of our findings. Nonetheless, future studies should aim to include larger and longitudinal cohorts to validate and extend our results. Second, although QSM is a highly sensitive technique for assessing iron deposition, its measurements can be affected by other paramagnetic or diamagnetic substances, such as calcium, copper, or lipids. The development of magnetic susceptibility source separation (χ-separation) techniques enables the independent quantification of positive and negative susceptibility sources, allowing for a more precise delineation of underlying susceptibility alterations.^69^ This approach has been successfully applied in studies of PD and Alzheimer’s disease.^70,71^ Consequently, future research incorporating χ-separation is warranted to disentangle the respective contributions of paramagnetic and diamagnetic sources among different PD motor subtypes. Third, the current analyses were cross-sectional, and the progression patterns were inferred from the baseline characteristics of PD subgroups with varying disease durations. Despite these limitations, our study provides preliminary evidence supporting distinct spatiotemporal patterns of nigrostriatal iron deposition across PD motor subtypes. Longitudinal studies in larger, independent cohorts are essential to confirm these observations and to elucidate the mechanisms underlying the dynamic modulation of nigrostriatal iron across PD motor subtypes.

## Conclusion

In conclusion, we identified distinct patterns of iron accumulation in the nigrostriatal system underlying different motor subtypes of PD, with an inverted U-shaped pattern observed in the PIGD subtype and an S-shaped pattern in the TD subtype. Moreover, iron distribution evolved along divergent trajectories between subtypes, specifically along the medial to lateral axis of the SN and the anterior to posterior axis of the putamen. These findings highlight subtype-specific iron dynamics and suggest that spatiotemporal iron deposition may underlie the divergent motor phenotypes in PD. Our results provide mechanistic insights into PD pathophysiology and may inform the development of targeted, subtype-specific interventions or disease-modifying therapies.

## Supplementary Material

fcaf473_Supplementary_Data

## Data Availability

The data that support the findings of this study are available from the corresponding author upon reasonable request. The code to generate QSM functions along the principal axes of a subcortical structure at the individual subject level can be downloaded from https://github.com/MezerLab/mrGrad.
