# Supplementary material for: Spatiotemporal nigrostriatal iron accumulation in motor subtypes of Parkinson’s disease: from early to late stage
Source: Brain Commun. 2025 Dec 3;7(6):fcaf473. doi: 10.1093/braincomms/fcaf473 (PMC12690202; doi:10.1093/braincomms/fcaf473)
Supplement: fcaf473_Supplementary_Data [file fcaf473_supplementary_data.pdf]

Supplementary material

Supplementary material 1. The SVD algorithm for orthogonal axis calculation

The SVD algorithm solves for

$$M = U\,\Sigma\,V^*$$

where M represents an m × 3 matrix of the ROI's m-centered image coordinates, Σ is a diagonal matrix containing the singular values of M, and U and V are matrices with columns representing M's left and right singular vectors, respectively. Consequently, the columns of the 3 × 3 matrix V define the principal orthogonal axes of the ROI based on its anatomical shape [1].

In the present study, three orthogonal axes of the bilateral SN, putamen, and caudate nucleus were identified as the anterior-posterior (AP), ventral-dorsal (VD), and medial-lateral (ML) axes. The directionality of the SVD-derived axes was determined as follows: the AP axis decreased with the y coordinate, the VD axis increased with the z coordinate, and the ML axis increased with the x coordinate in the right hemisphere and decreased with the x coordinate in the left hemisphere[1].

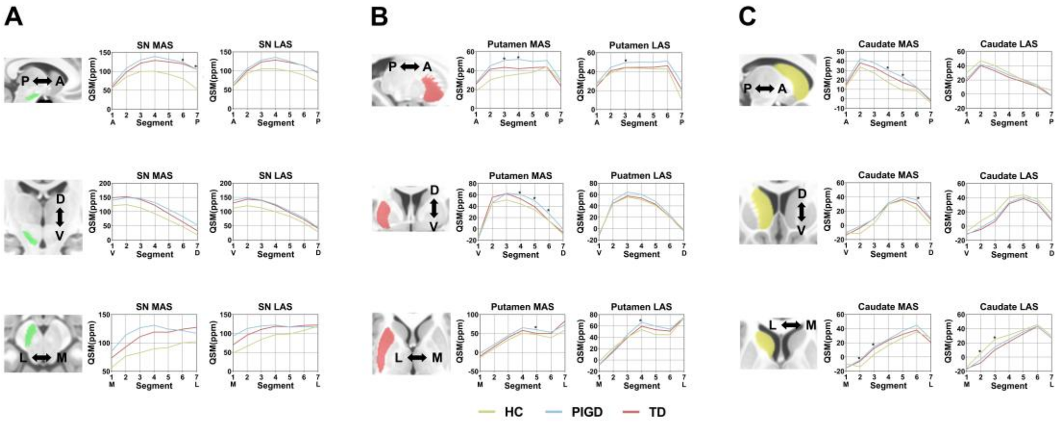

Supplementary Figure 1 Spatial iron deposition patterns in the nigrostriatal system across HC (yellow), PIGD (blue) and TD (red) subjects. N_{HC} = 47, N_{PIGD} = 55, N_{TD} = 53. Lines show mean QSM values of all subjects at each segment within the group. **(A)** gradients of SN iron deposition; **(B)** gradients of putamen iron deposition; **(C)** gradients of caudate iron deposition. Group differences were assessed by general linear models, adjusted for age, sex, and LEDD (for details, see Supplementary Table 2). Segment represents the specific section along the axis. A, anterior; D, Dorsal; HC, healthy controls; LAS, less affected side; L, lateral; MAS, more affected side; M, medial; PIGD, postural instability and gait difficulty subtype; P, posterior; SN, substantia nigra; TD, tremor-dominant subtype; V, Ventral. * denotes p value <0.05 among three groups.

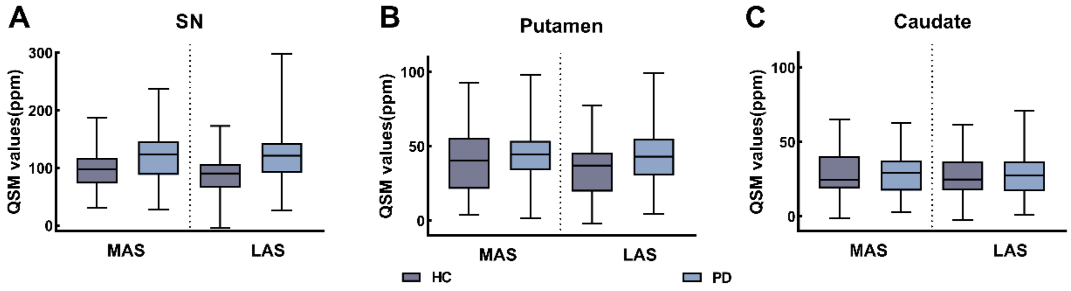

Supplementary Figure 2 comparisons of regional QSM values between HC (purple) and PD (blue) groups ($N_{HC} = 47$, $N_{PD} = 108$). Bars indicate mean QSM values ± SD for each group. **(A)** comparisons of SN iron deposition; **(B)** comparisons of putamen iron deposition; **(C)** comparisons of caudate iron deposition. Group differences were assessed by general linear models, adjusted for age, sex, and LEDD (for details, see Table 2). HC, healthy controls; LAS, less affected side; MAS, more affected side; PD, Parkinson's disease; QSM, quantitative susceptibility mapping; SD, standard deviation; SN, substantia nigra.

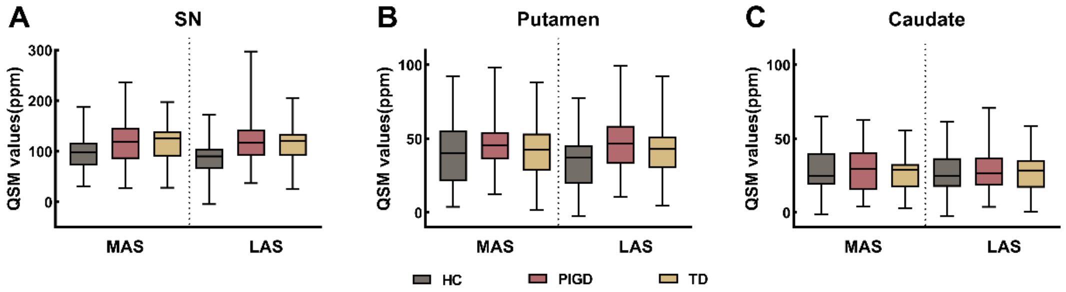

Supplementary Figure 3 Spatial iron deposition patterns in the nigrostriatal system across HC (grey), PIGD (red) and TD (yellow) subjects ($N_{HC} = 47$, $N_{PIGD} = 55$, $N_{TD} = 53$). Bars indicate mean QSM values ± SD for each group. **(A)** comparisons of SN iron deposition; **(B)** comparisons of putamen iron deposition; **(C)** comparisons of caudate iron deposition. Group differences were assessed by general linear models, adjusted for age, sex, and LEDD (for details, see Table 3). HC, healthy controls; LAS, less affected side; MAS, more affected side; PIGD, postural instability and gait difficulty subtype; QSM, quantitative susceptibility mapping; SD, standard deviation; SN, substantia nigra; TD, tremor-dominant subtype.

Supplementary Table 1 Status of all PD patients in scan

PD motor subtype	status
PIGD subtype	
sub2792	ON
sub2982	ON
sub3052	OFF
sub4072	ON
sub4132	ON
sub4382	ON
sub4422	ON
sub4442	ON
sub468	ON
sub470	ON
sub473	ON
sub474	ON
sub477	ON
sub4772	ON
sub480	OFF
sub493	ON
sub505	ON
sub5052	ON
sub508	ON
sub514	ON
sub5142	ON
sub517	ON
sub518	OFF
sub519	OFF
sub524	OFF
sub526	ON
sub533	OFF
sub534	ON
sub536	OFF
sub543	ON
sub546	ON
sub547	ON
sub553	ON
sub554	ON
sub560	ON
sub565	ON
sub568	OFF
sub570	ON
sub576	ON

sub578	ON
sub581	ON
sub583	ON
sub584	OFF
sub585	ON
sub590	ON
sub595	ON
sub598	ON
sub599	ON
sub601	ON
sub602	ON
sub609	OFF
sub619	ON
sub628	ON
sub635	ON
sub638	ON
TD subtype	
sub1032	ON
sub1162	ON
sub4222	ON
sub4332	ON
sub4372	ON
sub4392	OFF
sub4462	ON
sub4512	ON
sub479	ON
sub4792	ON
sub483	ON
sub4832	ON
sub484	OFF
sub488	ON
sub492	OFF
sub496	ON
sub4962	ON
sub498	OFF
sub499	ON
sub510	ON
sub511	ON
sub5112	ON
sub512	ON
sub5182	OFF
sub520	OFF
sub527	ON

sub528	ON
sub531	ON
sub532	ON
sub540	ON
sub545	ON
sub550	ON
sub552	ON
sub556	OFF
sub559	OFF
sub561	ON
sub573	OFF
sub579	OFF
sub588	OFF
sub593	ON
sub596	OFF
sub604	ON
sub606	ON
sub610	ON
sub611	ON
sub612	ON
sub615	ON
sub620	ON
sub621	ON
sub622	ON
sub623	ON
sub624	ON
sub637	OFF

Abbreviations: HC, healthy controls; PD, Parkinson's disease; PIGD, postural instability and gait difficulty subtype; TD, tremor-dominant subtype.

Supplementary Table 2 Differences in nigrostriatal iron deposition among HC, PIGD and TD groups.

Variable	HC (n = 47)	PIGD (n = 55)	TD (n = 53)	F	P	HC vs. PIGD	HC vs. TD	PIGD vs.TD
MAS-SN-AP-1	55.514± 8.908	65.042± 50.113	59.486 ± 43.35	0.418	0.659	1	1	1
MAS-SN-AP-2	85.996 ± 42.55	108.752± 57.255	98.573 ± 45.339	0.612	0.544	1	1	0.816
MAS-SN-AP-3	99.899 ± 40.586	130.165± 58.018	121.821± 43.008	0.414	0.662	1	1	1
MAS-SN-AP-4	100.444± 37.587	139.519 ± 62.44	129.247± 42.893	0.987	0.375	0.535	1	1
MAS-SN-AP-5	92.312 ± 35.114	131.799± 57.868	125.092± 38.872	2.031	0.135	0.146	0.35	1
MAS-SN-AP-6	80.738 ± 36.146	124.094± 45.203	120.082± 43.898	5.461	**0.005**	**0.006**	**0.015**	1
MAS-SN-AP-7	52.654 ± 38.501	104.679± 47.774	104.925± 50.741	9.178	**<0.001**	**<0.001**	**0.001**	1
LAS-SN-AP-1	59.466 ± 45.109	67.575 ± 44.788	60.357 ± 53.161	0.775	0.463	1	1	0.728
LAS-SN-AP-2	93.982 ± 45.072	105.941± 49.228	100.551± 52.341	0.585	0.558	1	1	1
LAS-SN-AP-3	105.215± 38.488	128.393± 50.243	123.556± 47.219	0.481	0.619	1	1	1
LAS-SN-AP-4	105.535± 37.756	135.858± 57.365	128.679± 47.064	0.529	0.591	1	1	1
LAS-SN-AP-5	98.957 ± 34.991	125.14 ± 51.546	122.145± 44.985	0.31	0.734	1	1	1
LAS-SN-AP-6	89.003 ± 33.522	113.35 ± 46.826	114.267± 47.637	0.397	0.673	1	1	1
LAS-SN-AP-7	71.758 ± 36.202	96.351 ± 53.406	94.453 ± 55.067	0.199	0.82	1	1	1
MAS-PUT-AP-1	18.219 ± 19.498	27.234 ± 16.654	25.803 ± 19.508	1.464	0.235	0.288	0.505	1
MAS-PUT-AP-2	30.282 ± 18.582	44.949 ± 18.457	41.442 ± 19.812	2.924	0.057	0.051	0.379	0.713
MAS-PUT-AP-3	34.186 ± 18.116	50.844 ± 22.995	43.417 ± 21.418	3.698	**0.027**	**0.037**	0.989	0.138
MAS-PUT-AP-4	36.335 ± 20.996	51.971 ± 24.831	41.983 ± 22.061	3.86	**0.023**	0.068	1	0.051
MAS-PUT-AP-5	38.23 ± 21.946	49.612 ± 27.095	43.107 ± 23.645	2.056	0.132	0.198	1	0.358
MAS-PUT-AP-6	44.05 ± 24.198	50.748 ± 37.689	43.734 ± 27.747	1.358	0.26	0.567	1	0.428
MAS-PUT-AP-7	27.866 ± 26.354	29.815 ± 46.866	23.636 ± 36.713	0.907	0.406	0.857	1	0.686
LAS-PUT-AP-1	27.074 ± 20.041	28.025 ± 22.105	24.329 ± 20.31	1.076	0.344	0.957	1	0.485
LAS-PUT-AP-2	38.787 ± 19.928	44.554 ± 21.146	41.009 ± 18.562	2.385	0.096	0.152	1	0.261
LAS-PUT-AP-3	43.845 ± 19.692	49.502 ± 23.724	44.311 ± 21.168	3.096	**0.048**	0.135	1	0.088
LAS-PUT-AP-4	43.703 ± 23.32	50.065 ± 22.64	44.526 ± 24.724	2.518	0.084	0.423	1	0.095
LAS-PUT-AP-5	42.93 ± 25.032	49.948 ± 23.394	44.636 ± 25.071	2.028	0.135	0.556	1	0.161
LAS-PUT-AP-6	42.724 ± 30.196	51.234 ± 31.651	46.192 ± 31.371	0.847	0.431	1	1	0.59
LAS-PUT-AP-7	10.249 ± 33.247	28.303 ± 41.739	20.808 ± 33.739	0.563	0.571	1	1	0.889
MAS-CAU-AP-1	13.063 ± 14.559	17.721 ± 16.654	15.135 ± 20.007	0.254	0.776	1	1	1
MAS-CAU-AP-2	33.635 ± 15.763	42.543 ± 19.536	38.092 ± 20.474	1.194	0.306	0.467	1	0.735
MAS-CAU-AP-3	27.317 ± 15.174	38.09 ± 17.746	33.07 ± 17.7	2.721	0.069	0.09	1	0.277
MAS-CAU-AP-4	17.206 ± 13.924	29.941 ± 17.015	24.931 ± 14.524	4.496	**0.013**	**0.013**	0.489	0.154
MAS-CAU-AP-5	9.075 ± 13.005	23.162 ± 15.973	17.409 ± 12.79	7.274	**0.001**	**0.001**	0.136	0.07
MAS-CAU-AP-6	8.574 ± 12.417	12.159 ± 15.926	11.817 ± 13.401	0.874	0.419	0.586	0.885	1
MAS-CAU-AP-7	-4.457 ± 11.339	-1.244 ± 14.243	-2.325 ± 13.408	0.004	0.996	1	1	1
LAS-CAU-AP-1	29.833 ± 20.253	21.438 ± 24.493	18.222 ± 20.596	0.725	0.486	1	0.797	1
LAS-CAU-AP-2	46.807 ± 19.931	42.004 ± 24.016	40.137 ± 23.159	0.244	0.784	1	1	1
LAS-CAU-AP-3	40.017 ± 17.227	35.633 ± 19.589	32.737 ± 21.06	0.902	0.408	1	0.967	0.695
LAS-CAU-AP-4	29.892 ± 15.547	26.818 ± 15.421	23.612 ± 19.077	2.014	0.137	1	0.233	0.4
LAS-CAU-AP-5	19.654 ± 14.39	20.638 ± 14.37	17.274 ± 17.374	1.667	0.193	1	1	0.217
LAS-CAU-AP-6	10.633 ± 12.883	12.828 ± 14.665	10.003 ± 14.094	1.805	0.168	0.454	1	0.246
LAS-CAU-AP-7	5.517 ± 12.232	-1.99 ± 12.038	-2.477 ± 11.5	0.775	0.463	1	0.648	1
MAS-SN-VD-1	121.12 ± 52.793	139.817± 70.272	148.732± 65.061	0.516	0.598	1	0.947	1
MAS-SN-VD-2	124.54 ± 48.481	149.139± 55.701	152.943± 48.758	0.568	0.568	1	0.928	1

MAS-SN-VD-3	113.35 ± 36.805	145.673± 52.913	142.622± 42.491	1.239	0.293	0.358	0.738	1
MAS-SN-VD-4	92.677 ± 34.293	131.89 ± 62.115	122.365± 42.439	1.457	0.236	0.278	0.994	1
MAS-SN-VD-5	69.347 ± 32.794	107.528± 78.577	91.976 ± 41.612	0.898	0.410	0.734	1	0.806
MAS-SN-VD-6	45.861 ± 34.382	81.751 ± 79.487	62.707 ± 41.982	1.048	0.353	0.856	1	0.538
MAS-SN-VD-7	17.724 ± 38.699	52.277 ± 73.603	32.57 ± 42.071	1.451	0.238	0.524	1	0.39
LAS-SN-VD-1	112.347± 50.251	139.734± 67.453	129.96 ± 66.419	1.042	0.355	0.459	1	1
LAS-SN-VD-2	120.345± 45.873	148.371± 55.633	143.659± 45.849	1.37	0.258	0.301	0.752	1
LAS-SN-VD-3	113.265± 37.744	140.677 ± 51.05	139.472± 47.906	0.909	0.405	0.552	0.903	1
LAS-SN-VD-4	99.28 ± 33.751	125.118 ± 53.57	121.926± 49.673	0.233	0.792	1	1	1
LAS-SN-VD-5	82.917 ± 34.298	101.678± 55.036	96.251 ± 48.179	0.217	0.805	1	1	1
LAS-SN-VD-6	59.496 ± 40.92	77.341 ± 51.283	71.955 ± 51.231	0.282	0.755	1	1	1
LAS-SN-VD-7	28.454 ± 47.848	43.159 ± 48.12	40.425 ± 55.65	0.203	0.816	1	1	1
MAS-PUT-VD-1	-13.717 ± 14.98	-18.22 ± 19.954	-14.924 ± 20.128	1.016	0.365	1	0.587	0.937
MAS-PUT-VD-2	45.487 ± 24.175	45.907 ± 28.2	55.589 ± 30.173	2.433	0.091	1	0.116	0.473
MAS-PUT-VD-3	51.277 ± 23.486	62.756 ± 24.252	62.219 ± 25.195	1.917	0.151	0.175	0.331	1
MAS-PUT-VD-4	44.14 ± 21.52	60.562 ± 26.42	52.96 ± 23.592	3.174	**0.045**	0.065	1	0.174
MAS-PUT-VD-5	33.331 ± 19.613	49.894 ± 27.761	38.868 ± 23.795	4.013	**0.020**	0.079	1	**0.035**
MAS-PUT-VD-6	14.195 ± 17.268	26.982 ± 25.015	14.211 ± 19.254	5.514	**0.005**	0.084	1	**0.005**
MAS-PUT-VD-7	-9.583 ± 11.974	-0.521 ± 16.764	-6.667 ± 12.174	2.72	0.069	0.119	1	0.187
LAS-PUT-VD-1	-9.204 ± 19.963	-13.563 ± 24.391	-11.095 ± 20.59	1.269	0.284	0.719	0.345	1
LAS-PUT-VD-2	44.016 ± 24.959	49.814 ± 33.127	44.682 ± 29.898	2.047	0.133	0.17	1	0.466
LAS-PUT-VD-3	55.719 ± 25.949	64.386 ± 26.121	57.568 ± 24.782	3.053	0.050	0.125	1	0.101
LAS-PUT-VD-4	51.079 ± 24.434	60.171 ± 22.429	53.901 ± 24.374	2.836	0.062	0.257	1	0.081
LAS-PUT-VD-5	41.249 ± 24.474	47.892 ± 22.145	42.886 ± 25.875	1.912	0.152	0.831	1	0.163
LAS-PUT-VD-6	20.285 ± 22.058	24.348 ± 19.653	20.856 ± 24.266	1.11	0.332	1	1	0.417
LAS-PUT-VD-7	-1.253 ± 16.304	-4.072 ± 13.748	-4.159 ± 16.371	1.072	0.345	0.77	0.45	1
MAS-CAU-VD-1	-10.352 ± 13.718	-10.128 ± 13.564	-13.732 ± 14.597	0.865	0.423	1	1	0.643
MAS-CAU-VD-2	-11.574 ± 15.152	-1.405 ± 18.199	-3.664 ± 20.036	1.02	0.363	0.578	0.583	1
MAS-CAU-VD-3	3.111 ± 14.953	9.083 ± 19.688	9.662 ± 23.378	0.129	0.879	1	1	1
MAS-CAU-VD-4	29.661 ± 18.618	31.605 ± 24.287	29.712 ± 24.929	0.244	0.784	1	1	1
MAS-CAU-VD-5	35.1 ± 14.769	40.813 ± 17.412	36.814 ± 18.489	0.758	0.471	1	1	0.686
MAS-CAU-VD-6	23.14 ± 14.594	35.824 ± 16.708	29.93 ± 16.354	4.876	**0.009**	**0.008**	0.38	0.148
MAS-CAU-VD-7	1.056 ± 11.003	9.857 ± 14.581	8.064 ± 13.299	2.763	0.066	0.067	0.187	1
LAS-CAU-VD-1	-7.615 ± 14.09	-11.852 ± 15.165	-10.9 ± 14.867	0.016	0.984	1	1	1
LAS-CAU-VD-2	7.346 ± 18.41	-2.185 ± 17.425	-4.737 ± 15.14	1.697	0.187	0.882	0.209	1
LAS-CAU-VD-3	18.698 ± 15.082	9.729 ± 21.26	6.606 ± 18.074	0.827	0.439	1	0.906	0.84
LAS-CAU-VD-4	40.76 ± 22.424	33.244 ± 28.536	31.515 ± 24.826	0.17	0.844	1	1	1
LAS-CAU-VD-5	43.946 ± 19.516	41.178 ± 22.238	38.729 ± 19.558	0.355	0.702	1	1	1
LAS-CAU-VD-6	37.787 ± 16.977	34.318 ± 20.141	30.908 ± 19.55	1.018	0.364	1	1	0.554
LAS-CAU-VD-7	16.947 ± 12.661	9.771 ± 12.745	7.804 ± 17.264	2.418	0.093	0.592	0.091	0.943
MAS-SN-ML-1	56.537 ± 42.048	85.117 ± 53.46	73.747 ± 54.113	0.710	0.493	1	1	0.722
MAS-SN-ML-2	77.029 ± 41.1	114.086± 84.776	92.946 ± 55.917	1.319	0.271	1	1	0.322
MAS-SN-ML-3	82.521 ± 37.486	126.523 ± 65.66	111.048 ± 50.243	2.027	0.136	0.206	1	0.361
MAS-SN-ML-4	89.856 ± 36.386	131.069± 57.202	119.117 ± 41.908	2.045	0.133	0.159	1	0.537
MAS-SN-ML-5	91.712 ± 38.169	124.174± 48.471	118.871± 40.765	1.191	0.307	0.38	0.762	1
MAS-SN-ML-6	100.323± 45.898	121.711± 47.878	124.121± 47.284	0.579	0.562	1	0.852	1
MAS-SN-ML-7	102.279± 53.193	116.927 ± 56.49	127.412± 57.062	1.157	0.317	1	0.516	0.811

LAS-SN-ML-1	48.147 ± 32.739	93.097 ± 89.941	70.026 ± 45.62	1.806	0.168	0.298	1	0.355
LAS-SN-ML-2	66.191 ± 37.363	112.92 ± 87.823	95.53 ± 54.098	1.083	0.342	0.589	1	0.696
LAS-SN-ML-3	84.283 ± 34.63	119.794± 61.529	110.673± 51.184	0.758	0.470	0.763	1	1
LAS-SN-ML-4	96.878 ± 35.064	122.183± 46.549	118.832± 45.843	0.447	0.640	1	1	1
LAS-SN-ML-5	98.395 ± 37.706	116.994 ± 44.38	117.246± 44.364	0.237	0.790	1	1	1
LAS-SN-ML-6	108.044± 45.991	118.027± 53.615	121.144± 48.724	0.125	0.883	1	1	1
LAS-SN-ML-7	119.097± 55.545	116.447± 67.809	122.183± 56.112	0.006	0.994	1	1	1
MAS-PUT-ML-1	-13.392 ± 13.923	-1.951 ± 26.445	-10.018 ± 19.613	1.230	0.295	0.573	1	0.534
MAS-PUT-ML-2	10.574 ± 18.815	20.956 ± 26.261	15.364 ± 22.997	2.057	0.132	0.139	0.746	0.774
MAS-PUT-ML-3	33.066 ± 20.974	44.985 ± 23.908	39.942 ± 23.899	3.049	0.051	0.047	0.486	0.503
MAS-PUT-ML-4	50.765 ± 23.65	65.062 ± 25.441	58.154 ± 23.268	2.645	0.075	0.125	1	0.202
MAS-PUT-ML-5	46.92 ± 21.787	59.636 ± 28.136	50.042 ± 21.776	3.332	**0.039**	0.378	1	**0.036**
MAS-PUT-ML-6	38.703 ± 20.578	53.051 ± 22.886	49.809 ± 19.331	1.688	0.189	0.233	1	0.683
MAS-PUT-ML-7	58.719 ± 26.736	72.969 ± 31.855	83.258 ± 36.866	2.448	0.090	1	0.113	0.481
LAS-PUT-ML-1	-2.422 ± 23.808	-7.955 ± 24.373	-8.552 ± 20.864	0.768	0.466	0.941	0.685	1
LAS-PUT-ML-2	21.652 ± 22.974	17.067 ± 26.559	14.674 ± 24.166	0.819	0.443	1	1	0.676
LAS-PUT-ML-3	40.678 ± 22.866	44.734 ± 23.154	39.887 ± 24.323	2.593	0.078	0.258	1	0.113
LAS-PUT-ML-4	53.408 ± 22.469	66.547 ± 23.792	59.353 ± 23.382	4.188	**0.017**	0.05	1	**0.041**
LAS-PUT-ML-5	44.712 ± 22.063	59.515 ± 25.245	53.261 ± 21.846	2.265	0.108	0.266	1	0.18
LAS-PUT-ML-6	44.517 ± 20.932	54.355 ± 21.402	50.962 ± 19.131	1.458	0.236	0.413	1	0.483
LAS-PUT-ML-7	74.608 ± 37.609	75.547 ± 33.044	74.769 ± 36.496	0.294	0.745	1	1	1
MAS-CAU-ML-1	-9.612 ± 14.601	-15.267 ± 15.541	-15.47 ± 16.192	0.258	0.773	1	1	1
MAS-CAU-ML-2	-13.747 ± 13.692	-4.172 ± 15.11	-5.91 ± 15.518	4.733	**0.010**	**0.012**	**0.024**	1
MAS-CAU-ML-3	3.673 ± 15.931	14.685 ± 16.765	12.386 ± 16.717	5.779	**0.004**	**0.004**	**0.013**	1
MAS-CAU-ML-4	16.351 ± 15.267	25.095 ± 14.941	23.557 ± 15.008	2.433	0.091	0.101	0.212	1
MAS-CAU-ML-5	26.756 ± 14.619	36.6 ± 17.255	31.86 ± 16.549	1.823	0.165	0.258	1	0.408
MAS-CAU-ML-6	35.103 ± 14.947	44.828 ± 21.497	37.906 ± 21.9	1.973	0.143	0.586	1	0.169
MAS-CAU-ML-7	27.052 ± 24.775	25.076 ± 30.188	20.546 ± 29.369	1.745	0.178	0.629	0.192	1
LAS-CAU-ML-1	-14.324 ± 15.401	-17.502 ± 17.856	-16.086 ± 15.716	0.026	0.974	1	1	1
LAS-CAU-ML-2	5.492 ± 17.365	-3.464 ± 16.921	-8.199 ± 17.463	4.271	**0.016**	0.467	**0.015**	0.309
LAS-CAU-ML-3	25.13 ± 17.058	14.355 ± 19.709	10.187 ± 17.34	3.381	**0.037**	0.665	**0.038**	0.407
LAS-CAU-ML-4	31.067 ± 14.622	25.13 ± 18.083	21.515 ± 16.552	1.837	0.163	1	0.316	0.386
LAS-CAU-ML-5	38.336 ± 15.987	35.705 ± 19.989	32.737 ± 18.592	0.871	0.421	1	1	0.601
LAS-CAU-ML-6	45.727 ± 23.32	43.567 ± 25.128	43.317 ± 25.329	0.058	0.944	1	1	1
LAS-CAU-ML-7	29.949 ± 26.504	27.246 ± 34.81	26.852 ± 29.92	0.177	0.838	1	1	1

P-values for statistically significant differences after Bonferroni correction are shown in bold; A = Anterior; CAU, caudate; D = Dorsal; HC, healthy controls; LAS, less affected side; L = Lateral; MAS, more affected side; M = Medial; P = Posterior; PIGD, postural instability and gait difficulty subtype; PUT, putamen; SN, Substantia nigra; TD, tremor-dominant subtype; V = Ventral.

Supplementary Table 3 Detailed data and statistical values for Figure 2

Supplementary Table 3A

Variable	HC(n=47)	PIGD-E(n=11)	PIGD-M(n=28)	PIGD-L(n=16)	F	P
MAS_Putamen	39.170±20.540	46.932±18.895	50.471±19.518	40.018 ± 17.066	1.685	0.526
LAS_Putamen	34.882±18.550	53.432±20.750	50.267±24.866	37.181±14.222	3.575	0.101
MAS_Caudate	28.059±14.516	29.078±10.258	31.972±17.227	26.491±15.556	0.970	0.673
LAS_Caudate	27.091±13.474	29.780±13.999	29.230±17.790	28.674±10.774	0.121	0.948
MAS_SN	95.629±33.086	105.696±44.463	122.032±48.454	120.233±31.624	0.600	0.739
LAS_SN	87.805±33.859	114.271±49.670	126.976±52.741	121.084±41.418	0.892	0.673

Supplementary Table 3B

Variable	HC(n=47)	TD-E(n=12)	TD-M(n=26)	TD-L(n=15)	F	P
MAS_Putamen	39.170±20.540	47.782±21.040	35.661±17.299	52.310±15.366	1.685	0.526
LAS_Putamen	34.882±18.550	48.059±17.530	36.768±21.596	42.979±12.676	3.575	0.101
MAS_Caudate	28.059±14.516	32.234±15.772	23.293±13.626	32.119±7.623	0.970	0.673
LAS_Caudate	27.091±13.474	29.118±15.641	23.372±14.310	30.339±5.977	0.121	0.948
MAS_SN	95.629±33.086	117.909±46.146	108.559±40.430	126.006±24.923	0.600	0.739
LAS_SN	87.805±33.859	120.731±38.548	111.325±39.364	120.795±15.901	0.892	0.673

Note: HC, healthy controls; LAS, less affected side; MAS, more affected side; PIGD, postural instability and gait difficulty subtype; PIGD-E, early disease stage of PIGD; PIGD-M, middle disease stage of PIGD; PIGD-L, late disease stage of PIGD; SN, Substantia nigra; TD, tremor-dominant subtype; TD-E, early disease stage of TD; TD-M, middle disease stage of TD; TD-L, late disease stage of TD.

Supplementary Table 4 Differences in nigrostriatal iron deposition among HC, and different disease stages of PIGD.

Variable	HC (n = 47)	PIGD-E (n = 11)	PIGD-M (n = 28)	PIGD-L (n = 16)	F	P	HC vs. PIGD-E	HC vs. PIGD-M	HC vs. PIGD-L	PIGD-E vs. PIGD-M	PIGD-E vs. PIGD-L	PIGD-M vs. PIGD-L
MAS-SN-AP-1	55.514± 38.908	87.948 ± 58.092	60.218 ± 44.069	57.735 ± 53.009	1.469	0.228	1	1	1	0.277	0.466	1
MAS-SN-AP-2	85.996 ± 42.55	122.598 ± 66.507	106.871 ± 54.452	102.524 ± 57.681	0.74	0.531	1	1	1	1	1	1
MAS-SN-AP-3	99.899 ± 40.586	124.388 ± 64.25	133.247 ± 61.07	128.743 ± 51.072	0.174	0.914	1	1	1	1	1	1
MAS-SN-AP-4	100.444 ± 37.587	122.006 ± 57.19	146.09 ± 70.572	140.061 ± 50.836	0.601	0.616	1	1	1	1	1	1
MAS-SN-AP-5	92.312 ± 35.114	110.148 ± 49.038	140.718 ± 66.248	131.076 ± 45.382	1.573	0.201	1	0.262	1	0.926	1	1
MAS-SN-AP-6	80.738 ± 36.146	106.157 ± 55.151	129.753 ± 42.03	126.522 ± 42.971	3.742	**0.014**	1	**0.007**	0.118	0.627	1	1
MAS-SN-AP-7	52.654 ± 38.501	88.127 ± 62.637	109.051 ± 44.893	108.408 ± 41.495	5.862	**0.001**	0.311	**<0.001**	**0.009**	0.506	0.663	1
LAS-SN-AP-1	59.466 ± 45.109	79.6 ± 44.486	67.375 ± 48.284	59.66 ± 39.153	0.483	0.695	1	1	1	1	1	1
LAS-SN-AP-2	93.982 ± 45.072	115.839 ± 48.538	104.81 ± 52.522	101.115 ± 45.707	0.328	0.805	1	1	1	1	1	1
LAS-SN-AP-3	105.215 ± 38.488	122.157 ± 46.094	129.826 ± 59.255	130.172 ± 36.27	0.255	0.857	1	1	1	1	1	1
LAS-SN-AP-4	105.535 ± 37.756	115.308 ± 47.616	139.656 ± 69.384	143.34 ± 35.686	0.772	0.512	1	1	1	1	1	1
LAS-SN-AP-5	98.957 ± 34.991	100.931 ± 51.403	130.805 ± 57.799	131.868 ± 35.389	1.265	0.291	1	0.994	1	0.413	0.853	1
LAS-SN-AP-6	89.003 ± 33.522	93.415 ± 61.404	119.418 ± 45.933	116.437 ± 34.813	1.138	0.338	1	0.954	1	0.539	1	1
LAS-SN-AP-7	71.758 ± 36.202	79.97 ± 68.097	101.45 ± 51.832	98.69 ± 45.711	0.601	0.616	1	1	1	1	1	1
MAS-PUT-AP-1	18.219 ± 19.498	28.877 ± 20.37	28.741 ± 17.827	23.467 ± 11.335	1.466	0.229	0.788	0.848	1	1	1	1
MAS-PUT-AP-2	30.282 ± 18.582	50.907 ± 12.912	46.655 ± 23.042	37.866 ± 8.637	3.834	**0.012**	0.080	0.564	1	1	0.163	0.276
MAS-PUT-AP-3	34.186 ± 18.116	60.406 ± 20.492	52.611 ± 26.782	41.176 ± 12.651	4.929	**0.003**	**0.033**	0.979	1	0.876	**0.025**	0.126
MAS-PUT-AP-4	36.335 ± 20.996	62.769 ± 29.225	53.567 ± 25.226	41.755 ± 17.418	4.028	**0.010**	0.078	1	1	0.751	**0.033**	0.219
MAS-PUT-AP-5	38.23 ± 21.946	56.563 ± 28.964	52.96 ± 27.614	38.975 ± 23.107	2.516	0.063	0.588	1	1	1	0.261	0.267
MAS-PUT-AP-6	44.05 ± 24.198	52.225 ± 40.047	57.697 ± 39.489	37.574 ± 30.997	1.606	0.193	1	1	1	1	1	0.388
MAS-PUT-AP-7	27.866 ± 26.354	23.504 ± 44.437	40.009 ± 52.792	16.313 ± 34.007	1.647	0.184	1	0.683	1	0.894	1	0.644
LAS-PUT-AP-1	27.074 ± 20.041	24.25 ± 17.209	30.051 ± 26.69	27.075 ± 16.131	1.175	0.323	1	0.565	1	0.925	1	1
LAS-PUT-AP-2	38.787 ± 19.928	43.627 ± 13.965	48.896 ± 25.081	37.592 ± 16.121	2.534	0.062	1	0.060	1	0.857	1	1
LAS-PUT-AP-3	43.845 ± 19.692	50.47 ± 16.647	55.84 ± 26.022	37.746 ± 19.988	3.565	**0.017**	1	**0.040**	1	0.811	1	0.252
LAS-PUT-AP-4	43.703 ± 23.32	51.922 ± 21.02	54.794 ± 23.87	40.511 ± 19.584	1.68	0.176	1	0.524	1	1	1	0.643
LAS-PUT-AP-5	42.93 ± 25.032	51.745 ± 26.307	52.812 ± 22.583	43.7 ± 23.053	0.553	0.647	1	1	1	1	1	1
LAS-PUT-AP-6	42.724 ± 30.196	55.168 ± 40.376	51.442 ± 30.873	48.166 ± 27.972	0.181	0.909	1	1	1	1	1	1
LAS-PUT-AP-7	10.249 ± 33.247	27.121 ± 41.069	27.286 ± 47.829	30.897 ± 31.928	0.044	0.988	1	1	1	1	1	1
MAS-CAU-AP-1	13.063 ± 14.559	20.04 ± 15.376	16.112 ± 17.814	18.941 ± 16.101	0.468	0.705	1	1	1	1	1	1
MAS-CAU-AP-2	33.635 ± 15.763	46.191 ± 17.214	41.686 ± 22.119	41.535 ± 16.839	0.823	0.484	0.797	1	1	1	1	1
MAS-CAU-AP-3	27.317 ± 15.174	39.656 ± 17.49	36.868 ± 19.48	39.153 ± 15.523	1.519	0.214	0.492	0.605	0.565	1	1	1
MAS-CAU-AP-4	17.206 ± 13.924	31.335 ± 18.572	29.035 ± 17.74	30.567 ± 15.572	2.465	0.067	0.195	0.16	0.219	1	1	1
MAS-CAU-AP-5	9.075 ± 13.005	22.635 ± 16.787	21.425 ± 16.188	26.566 ± 15.514	4.216	**0.008**	0.077	**0.027**	**0.014**	1	1	1

MAS-CAU-AP-6	8.574 ± 12.417	12.519 ± 17.985	10.92 ± 17.806	14.077 ± 10.951	0.393	0.759	1	1	1	1	1	1
MAS-CAU-AP-7	-4.457 ± 11.339	0.414 ± 16.691	-2.835 ± 13.633	0.401 ± 14.157	0.928	0.430	1	1	1	0.805	1	1
LAS-CAU-AP-1	29.833 ± 20.253	14.282 ± 20.093	28.94 ± 26.965	13.23 ± 19.162	2.238	0.089	1	1	1	0.217	1	0.404
LAS-CAU-AP-2	46.807 ± 19.931	41.554 ± 17.637	46.001 ± 27.065	35.318 ± 21.744	0.683	0.565	1	1	1	1	1	1
LAS-CAU-AP-3	40.017 ± 17.227	37.735 ± 16.205	37.378 ± 20.975	31.133 ± 19.592	0.183	0.908	1	1	1	1	1	1
LAS-CAU-AP-4	29.892 ± 15.547	28.345 ± 16.331	27.019 ± 16.035	25.414 ± 14.547	0.073	0.974	1	1	1	1	1	1
LAS-CAU-AP-5	19.654 ± 14.39	19.932 ± 15.35	20.467 ± 15.071	21.422 ± 13.281	0.7	0.554	1	1	1	1	1	1
LAS-CAU-AP-6	10.633 ± 12.883	15.537 ± 15.85	12.57 ± 14.497	11.416 ± 14.858	0.859	0.466	1	0.903	1	1	1	1
LAS-CAU-AP-7	5.517 ± 12.232	-0.569 ± 12.288	-0.753 ± 12.799	-5.133 ± 10.549	0.217	0.885	1	1	1	1	1	1
MAS-SN-VD-1	121.12 ± 52.793	114.985 ± 60.032	146.905 ± 71.851	144.484 ± 74.25	0.973	0.409	1	1	1	0.704	1	1
MAS-SN-VD-2	124.54 ± 48.481	130.41 ± 44.015	153.739 ± 57.079	153.965 ± 60.761	1.096	0.355	1	0.802	1	0.848	1	1
MAS-SN-VD-3	113.35 ± 36.805	135.99 ± 52.386	148.149 ± 55.148	147.997 ± 51.858	1.145	0.335	1	0.436	0.855	1	1	1
MAS-SN-VD-4	92.677 ± 34.293	125.371 ± 64.775	135.225 ± 69.191	130.538 ± 49.209	0.723	0.541	1	1	1	1	1	1
MAS-SN-VD-5	69.347 ± 32.794	102.063 ± 66.628	113.403 ± 94.926	101.006 ± 54.01	0.567	0.638	1	1	1	1	1	1
MAS-SN-VD-6	45.861 ± 34.382	80.829 ± 72.968	86.81 ± 94.425	73.534 ± 55.018	0.707	0.550	1	1	1	1	1	1
MAS-SN-VD-7	17.724 ± 38.699	56.391 ± 71.953	55.755 ± 85.152	43.361 ± 53.668	0.882	0.453	1	1	1	1	1	1
LAS-SN-VD-1	112.347 ± 50.251	113.866 ± 58.196	147.169 ± 70.387	144.507 ± 67.72	2.159	0.098	1	0.109	0.435	0.437	0.801	1
LAS-SN-VD-2	120.345 ± 45.873	129.44 ± 53.097	153.206 ± 61.291	152.924 ± 46.608	1.472	0.227	1	0.291	0.736	0.934	1	1
LAS-SN-VD-3	113.265 ± 37.744	126.13 ± 49.892	145.294 ± 59.735	142.598 ± 33.178	0.900	0.444	1	0.723	1	1	1	1
LAS-SN-VD-4	99.28 ± 33.751	113.683 ± 54.641	129.199 ± 62.54	125.838 ± 34.247	0.309	0.819	1	1	1	1	1	1
LAS-SN-VD-5	82.917 ± 34.298	95.569 ± 48.649	103.636 ± 67.155	102.451 ± 34.259	0.047	0.986	1	1	1	1	1	1
LAS-SN-VD-6	59.496 ± 40.92	76.256 ± 45.215	74.735 ± 60.748	82.646 ± 37.664	0.193	0.901	1	1	1	1	1	1
LAS-SN-VD-7	28.454 ± 47.848	49.54 ± 50.679	37.127 ± 51.698	49.329 ± 40.92	0.499	0.684	1	1	1	1	1	1
MAS-PUT-VD-1	-13.717 ± 14.98	-22.925 ± 25.903	-16.673 ± 17.801	-17.693 ± 19.865	0.35	0.789	1	1	1	1	1	1
MAS-PUT-VD-2	45.487 ± 24.175	40.415 ± 31.096	53.907 ± 29.873	35.682 ± 18.943	2.515	0.063	1	0.859	1	0.793	1	0.109
MAS-PUT-VD-3	51.277 ± 23.486	64.847 ± 17.499	67.74 ± 29.428	52.594 ± 13.953	2.91	**0.038**	1	0.65	1	1	0.639	0.106
MAS-PUT-VD-4	44.14 ± 21.52	69.268 ± 25.966	63.137 ± 28.974	50.07 ± 19.157	3.525	**0.018**	0.139	0.976	1	1	0.132	0.237
MAS-PUT-VD-5	33.331 ± 19.613	60.403 ± 30.821	51.919 ± 29.074	39.123 ± 20.18	3.668	**0.015**	0.090	1	1	1	0.085	0.294
MAS-PUT-VD-6	14.195 ± 17.268	36.122 ± 25.134	28.821 ± 27.352	17.478 ± 17.919	3.115	**0.030**	0.131	1	1	1	0.161	0.472
MAS-PUT-VD-7	-9.583 ± 11.974	1.719 ± 22.352	-0.765 ± 16.57	-1.632 ± 13.39	1.157	0.330	0.49	1	1	1	1	1
LAS-PUT-VD-1	-9.204 ± 19.963	-20.58 ± 21.206	-9.276 ± 26.24	-16.24 ± 22.902	1.838	0.146	1	0.303	1	0.254	0.745	1
LAS-PUT-VD-2	44.016 ± 24.959	41.117 ± 38.815	58.8 ± 29.409	40.069 ± 32.893	3.912	**0.011**	1	**0.017**	1	0.100	1	0.681
LAS-PUT-VD-3	55.719 ± 25.949	60.638 ± 28.793	70.871 ± 25.27	55.613 ± 24.15	2.827	**0.043**	1	0.056	1	0.489	1	0.874
LAS-PUT-VD-4	51.079 ± 24.434	61.03 ± 21.138	63.924 ± 23.028	53.013 ± 21.844	1.344	0.265	1	0.512	1	1	1	1
LAS-PUT-VD-5	41.249 ± 24.474	53.252 ± 20.038	50.337 ± 23.758	39.928 ± 19.545	0.811	0.491	1	1	1	1	1	1
LAS-PUT-VD-6	20.285 ± 22.058	30.958 ± 18.103	24.934 ± 22.274	18.779 ± 14.6	0.429	0.733	1	1	1	1	1	1
LAS-PUT-VD-7	-1.253 ±	-7.703 ±	-4.016 ±	-1.674 ±	1.31	0.276	0.438	1	1	1	1	1

	16.304	14.924	13.838	13.096									
MAS-CAU-VD-1	-10.352 ± 13.718	-11.739 ± 16.54	-10.746 ± 12.41	-7.94 ± 13.964	0.137	0.938	1	1	1	1	1	1
MAS-CAU-VD-2	-11.574 ± 15.152	-0.329 ± 17.947	-3.232 ± 19.129	1.053 ± 17.478	0.77	0.514	0.911	1	1	1	1	1
MAS-CAU-VD-3	3.111 ± 14.953	11.912 ± 19.399	7.898 ± 22.124	9.212 ± 15.968	1.77	0.158	1	0.605	0.354	0.566	0.261	1
MAS-CAU-VD-4	29.661 ± 18.618	34.954 ± 23.085	30.999 ± 27.478	30.362 ± 19.962	0.818	0.487	1	1	0.987	1	1	1
MAS-CAU-VD-5	35.1 ± 14.769	42.299 ± 14.398	40.58 ± 20.939	40.198 ± 12.726	0.247	0.863	1	1	1	1	1	1
MAS-CAU-VD-6	23.14 ± 14.594	38.345 ± 16.218	34.951 ± 18.909	35.619 ± 13.411	2.876	**0.040**	0.074	0.128	0.335	1	1	1
MAS-CAU-VD-7	1.056 ± 11.003	11.52 ± 14.175	6.365 ± 11.526	14.826 ± 18.47	2.591	0.057	0.249	1	0.155	1	1	0.319
LAS-CAU-VD-1	-7.615 ± 14.09	-11.954 ± 20.378	-10.642 ± 14.917	-13.901 ± 11.971	0.216	0.885	1	1	1	1	1	1
LAS-CAU-VD-2	7.346 ± 18.41	-2.299 ± 20.035	-0.604 ± 18.244	-4.873 ± 14.628	0.278	0.841	1	1	1	1	1	1
LAS-CAU-VD-3	18.698 ± 15.082	5.228 ± 20.077	14.331 ± 21.413	4.768 ± 21.27	1.233	0.302	1	1	1	0.633	1	1
LAS-CAU-VD-4	40.76 ± 22.424	27.903 ± 23.102	40.657 ± 30.483	23.945 ± 26.219	1.641	0.185	1	1	1	0.599	1	0.52
LAS-CAU-VD-5	43.946 ± 19.516	40.581 ± 15.958	45.459 ± 25.463	34.098 ± 18.857	0.975	0.408	1	1	1	1	1	0.989
LAS-CAU-VD-6	37.787 ± 16.977	34.147 ± 18.073	37.597 ± 22.105	28.697 ± 17.584	0.71	0.548	1	1	1	1	1	1
LAS-CAU-VD-7	16.947 ± 12.661	9.603 ± 11.996	9.463 ± 13.565	10.424 ± 12.529	0.821	0.486	1	1	1	1	1	1
MAS-SN-ML-1	56.537 ± 42.048	72.291 ± 44.183	85.739 ± 60.936	92.845 ± 46.025	0.065	0.978	1	1	1	1	1	1
MAS-SN-ML-2	77.029 ± 41.1	101.887 ± 68.547	118.46 ± 104.92	114.818 ± 52.73	0.094	0.963	1	1	1	1	1	1
MAS-SN-ML-3	82.521 ± 37.486	117.596 ± 64.816	128.917 ± 73.07	128.47 ± 55.098	0.516	0.672	1	1	1	1	1	1
MAS-SN-ML-4	89.856 ± 36.386	126.492 ± 63.114	133.05 ± 59.535	130.749 ± 52.164	0.819	0.487	1	1	1	1	1	1
MAS-SN-ML-5	91.712 ± 38.169	116.018 ± 50.413	128.353 ± 51.156	122.469 ± 44.37	0.857	0.466	1	0.789	1	1	1	1
MAS-SN-ML-6	100.323 ± 45.898	110.907 ± 39.874	128.615 ± 53.854	117.059 ± 42.12	0.719	0.543	1	1	1	1	1	1
MAS-SN-ML-7	102.279 ± 53.193	99.17 ± 43.562	127.585 ± 63.966	110.483 ± 48.687	0.981	0.405	1	1	1	1	1	1
LAS-SN-ML-1	48.147 ± 32.739	81.553 ± 69.11	94.733 ± 111.87	98.168 ± 57.278	0.32	0.811	1	1	1	1	1	1
LAS-SN-ML-2	66.191 ± 37.363	98.467 ± 69.734	113.469 ± 108.657	121.894 ± 55.075	0.101	0.959	1	1	1	1	1	1
LAS-SN-ML-3	84.283 ± 34.63	109.068 ± 64.956	120.301 ± 69.006	126.28 ± 45.992	0.112	0.953	1	1	1	1	1	1
LAS-SN-ML-4	96.878 ± 35.064	113.974 ± 55.603	123.283 ± 47.924	125.9 ± 39.229	0.228	0.877	1	1	1	1	1	1
LAS-SN-ML-5	98.395 ± 37.706	108.128 ± 46.75	119.963 ± 47.709	117.896 ± 38.23	0.451	0.717	1	1	1	1	1	1
LAS-SN-ML-6	108.044 ± 45.991	106.68 ± 41.948	124.294 ± 61.116	114.859 ± 47.682	0.808	0.493	1	1	1	1	1	1
LAS-SN-ML-7	119.097 ± 55.545	101.339 ± 50.026	125.595 ± 75.66	110.824 ± 64.989	0.888	0.450	1	1	1	0.729	1	1
MAS-PUT-ML-1	-13.392 ± 13.923	4.044 ± 34.365	-3.463 ± 29.384	-3.428 ± 11.963	1.636	0.186	0.301	1	1	0.631	0.501	1
MAS-PUT-ML-2	10.574 ± 18.815	31.966 ± 24.135	21.618 ± 28.881	12.229 ± 20.542	3.733	**0.014**	0.059	1	1	0.55	**0.047**	0.474
MAS-PUT-ML-3	33.066 ± 20.974	53.697 ± 19.409	48.511 ± 26.285	32.824 ± 18.065	4.421	**0.006**	0.106	0.61	1	1	0.077	0.084
MAS-PUT-ML-4	50.765 ± 23.65	69.357 ± 25.34	69.113 ± 35.537	53.269 ± 16.729	3.143	**0.029**	0.764	0.683	1	1	0.408	0.098
MAS-PUT-ML-5	46.92 ± 21.787	62.177 ± 29.721	63.914 ± 30.999	50.4 ± 20.035	1.642	0.185	1	1	1	1	1	0.442
MAS-PUT-ML-6	38.703 ± 20.578	56.425 ± 19.304	53.682 ± 27.335	49.628 ± 16.451	1.001	0.396	1	1	1	1	1	1
MAS-PUT-ML-7	58.719 ± 26.736	77.708 ± 27.032	70.113 ± 28.014	76.46 ± 28.906	0.535	0.659	1	1	1	1	1	1
LAS-PUT-ML-1	-2.422 ± 23.808	-12.062 ± 25.19	-9.31 ± 21.202	-2.76 ± 29.354	0.848	0.471	1	1	1	1	1	1

LAS-PUT-ML-2	21.652 ± 22.974	15.586 ± 29.501	19.96 ± 26.534	13.024 ± 25.637	0.419	0.740	1	1	1	1	1	1
LAS-PUT-ML-3	40.678 ± 22.866	45.666 ± 23.218	49.672 ± 23.634	35.452 ± 20.665	1.767	0.159	1	0.306	1	1	1	0.971
LAS-PUT-ML-4	53.408 ± 22.469	68.856 ± 23.39	71.237 ± 24.413	56.754 ± 21.285	2.735	**0.048**	0.996	0.146	1	1	1	0.431
LAS-PUT-ML-5	44.712 ± 22.063	62.98 ± 25.586	61.846 ± 28.616	53.054 ± 17.904	1.383	0.253	1	1	1	1	1	1
LAS-PUT-ML-6	44.517 ± 20.932	53.52 ± 21.505	57.783 ± 24.706	48.93 ± 13.714	1.452	0.233	1	0.38	1	1	1	1
LAS-PUT-ML-7	74.608 ± 37.609	65.749 ± 31.583	82.224 ± 39.511	70.599 ± 16.615	1.709	0.170	1	0.352	1	0.3	1	1
MAS-CAU-ML-1	-9.612 ± 14.601	-15.26 ± 16.582	-13.93 ± 17.921	-17.612 ± 9.946	0.725	0.540	1	1	1	1	1	1
MAS-CAU-ML-2	-13.747 ± 13.692	1.078 ± 18.511	-6.749 ± 13.416	-3.273 ± 15.363	3.624	**0.016**	**0.011**	0.506	0.377	0.790	1	1
MAS-CAU-ML-3	3.673 ± 15.931	19.325 ± 14.907	13.249 ± 17.905	14.009 ± 16.325	3.479	**0.019**	**0.018**	0.176	0.524	1	1	1
MAS-CAU-ML-4	16.351 ± 15.267	29.162 ± 13.611	23.665 ± 16.606	24.802 ± 12.95	1.54	0.209	0.214	1	1	1	1	1
MAS-CAU-ML-5	26.756 ± 14.619	39.484 ± 17.309	34.957 ± 19.345	37.492 ± 13.655	0.925	0.432	0.599	1	1	1	1	1
MAS-CAU-ML-6	35.103 ± 14.947	44.822 ± 21.2	44.299 ± 23.838	45.757 ± 18.431	0.443	0.723	1	1	1	1	1	1
MAS-CAU-ML-7	27.052 ± 24.775	17.274 ± 22.763	25.313 ± 28.637	30.023 ± 37.262	0.975	0.408	0.729	1	1	1	1	1
LAS-CAU-ML-1	-14.324 ± 15.401	-13.885 ± 28.038	-17.575 ± 14.396	-19.861 ± 15.336	0.119	0.949	1	1	1	1	1	1
LAS-CAU-ML-2	5.492 ± 17.365	-4.978 ± 21.271	-1.77 ± 18.059	-5.387 ± 11.448	0.97	0.410	0.589	1	1	1	1	1
LAS-CAU-ML-3	25.13 ± 17.058	9.487 ± 21.882	18.577 ± 21.106	10.314 ± 14.465	1.65	0.183	0.367	1	1	0.317	1	1
LAS-CAU-ML-4	31.067 ± 14.622	22.651 ± 15.935	28.673 ± 20.376	20.634 ± 14.53	1.039	0.379	1	1	1	0.649	1	1
LAS-CAU-ML-5	38.336 ± 15.987	35.666 ± 17.159	39.399 ± 22.002	29.269 ± 17.354	0.999	0.397	1	1	1	1	1	1
LAS-CAU-ML-6	45.727 ± 23.32	44.536 ± 18.352	46.918 ± 27.209	37.036 ± 25.471	0.507	0.678	1	1	1	1	1	1
LAS-CAU-ML-7	29.949 ± 26.504	29.006 ± 27.808	27.013 ± 38.709	26.445 ± 33.863	0.114	0.952	1	1	1	1	1	1

P-values for statistically significant differences after Bonferroni correction are shown in bold; A = Anterior; CAU, caudate; D = Dorsal; HC, healthy controls; LAS, less affected side; L = Lateral; MAS, more affected side; M = Medial; P = Posterior; PIGD, postural instability and gait difficulty subtype; PIGD-E, early disease stage of PIGD; PIGD-M, middle disease stage of PIGD; PIGD-L, late disease stage of PIGD; PUT, putamen; SN, Substantia nigra; V = Ventral.

Supplementary Table 5 Differences in nigrostriatal iron deposition among HC, and different disease stages of TD.

Variable	HC (n = 47)	TD-E (n = 12)	TD-M (n = 26)	TD-L (n = 15)	F	P	HC vs. TD-E	HC vs. TD-M	HC vs. TD-L	TD-E vs. TD-M	TD-E vs.TD-L	TD-M vs.TD-L
MAS-SN-AP-1	55.514 ± 38.908	67.956 ± 45.086	52.63 ± 43.159	70.431 ± 27.821	0.319	0.812	1	1	1	1	1	1
MAS-SN-AP-2	85.996 ± 42.55	107.784 ± 39.526	92.243 ± 51.068	106.296 ± 19.975	0.269	0.847	1	1	1	1	1	1
MAS-SN-AP-3	99.899 ± 40.586	130.637 ± 44.749	115.988 ± 45.414	128.371 ± 20.144	0.17	0.916	1	1	1	1	1	1
MAS-SN-AP-4	100.444 ± 37.587	136.887 ± 43.713	124.385 ± 45.867	134.21 ± 20.438	0.762	0.518	1	1	1	1	1	1
MAS-SN-AP-5	92.312 ± 35.114	129.426 ± 39.914	122.176 ± 41.939	128.493 ± 17.848	1.742	0.164	0.351	0.351	0.773	1	1	1
MAS-SN-AP-6	80.738 ± 36.146	115.893 ± 44.407	120.344 ± 47.806	126.289 ± 19.784	3.054	**0.032**	0.408	**0.041**	0.133	1	1	1
MAS-SN-AP-7	52.654 ± 38.501	94.275 ± 47.834	107.179 ± 55.762	114.812 ± 24.21	6.29	**0.001**	0.222	**0.001**	**0.009**	0.96	0.67	1
LAS-SN-AP-1	59.466 ± 45.109	67.974 ± 45.129	46.85 ± 46.887	97.466 ± 47.737	1.042	0.378	1	1	1	1	1	0.533
LAS-SN-AP-2	93.982 ± 45.072	114.63 ± 48.877	87.624 ± 50.395	124.429 ± 37.85	0.611	0.610	1	1	1	1	1	1
LAS-SN-AP-3	105.215 ± 38.488	130.844 ± 53.061	116.019 ± 45.4	139.061 ± 29.04	0.388	0.762	1	1	1	1	1	1
LAS-SN-AP-4	105.535 ± 37.756	129.027 ± 51.361	125.474 ± 47.507	139.985 ± 27.851	0.559	0.643	1	1	1	1	1	1
LAS-SN-AP-5	98.957 ± 34.991	124.835 ± 46.83	118.871 ± 45.921	129.691 ± 28.577	0.635	0.594	1	1	1	1	1	1
LAS-SN-AP-6	89.003 ± 33.522	115.845 ± 46.522	114.04 ± 50.77	112.41 ± 28.719	0.71	0.548	1	1	1	1	1	1
LAS-SN-AP-7	71.758 ± 36.202	89.87 ± 54.146	97.232 ± 59.158	91.985 ± 30.692	0.665	0.576	1	1	1	1	1	1
MAS-PUT-AP-1	18.219 ± 19.498	34.057 ± 19.472	20.418 ± 19.497	31.658 ± 9.487	1.505	0.219	0.563	1	1	0.33	1	1
MAS-PUT-AP-2	30.282 ± 18.582	50.234 ± 18.955	36.13 ± 19.706	46.1 ± 11.331	1.437	0.237	0.386	1	1	0.529	1	1
MAS-PUT-AP-3	34.186 ± 18.116	49.403 ± 17.736	39.565 ± 23.323	47.465 ± 12.518	0.657	0.581	1	1	1	1	1	1
MAS-PUT-AP-4	36.335 ± 20.996	48.252 ± 19.141	38.548 ± 23.463	43.995 ± 14.179	0.297	0.827	1	1	1	1	1	1
MAS-PUT-AP-5	38.23 ± 21.946	50.514 ± 18.681	40.207 ± 25.595	41.181 ± 15.767	0.477	0.699	1	1	1	1	1	1
MAS-PUT-AP-6	44.05 ± 24.198	49.811 ± 25.9	41.312 ± 28.757	42.316 ± 19.297	0.073	0.974	1	1	1	1	1	1
MAS-PUT-AP-7	27.866 ± 26.354	26.935 ± 34.309	19.125 ± 35.188	34.739 ± 31.487	0.832	0.480	1	1	1	1	1	0.77
LAS-PUT-AP-1	27.074 ± 20.041	33.506 ± 19.162	17.034 ± 19.795	35.693 ± 8.521	2.407	0.072	1	0.736	1	0.315	1	0.185
LAS-PUT-AP-2	38.787 ± 19.928	50.542 ± 18.869	34.19 ± 15.916	49.997 ± 12.09	1.703	0.172	1	1	1	0.453	1	0.445
LAS-PUT-AP-3	43.845 ± 19.692	52.237 ± 21.927	37.726 ± 17.797	55.183 ± 16.473	1.16	0.329	1	1	1	1	1	0.633
LAS-PUT-AP-4	43.703 ± 23.32	49.537 ± 25.447	37.998 ± 21.768	60.183 ± 18.522	1.121	0.345	1	1	1	1	1	0.427
LAS-PUT-AP-5	42.93 ± 25.032	50.873 ± 26.162	38.505 ± 22.752	56.713 ± 18.365	0.78	0.508	1	1	1	1	1	1
LAS-PUT-AP-6	42.724 ± 30.196	51.113 ± 34.298	43.316 ± 27.5	48.44 ± 27.924	0.075	0.973	1	1	1	1	1	1
LAS-PUT-AP-7	10.249 ± 33.247	17.153 ± 29.205	21.726 ± 30.504	23.662 ± 35.053	0.315	0.814	1	1	1	1	1	1
MAS-CAU-AP-1	13.063 ± 14.559	18.84 ± 22.057	12.477 ± 20.209	18.654 ± 10.745	0.693	0.559	1	1	1	1	1	1
MAS-CAU-AP-2	33.635 ± 15.763	39.021 ± 22.404	35.513 ± 20.402	46.082 ± 11.606	0.385	0.764	1	1	1	1	1	1
MAS-CAU-AP-3	27.317 ± 15.174	32.186 ± 18.855	31.609 ± 17.634	40.01 ± 11.015	0.505	0.680	1	1	1	1	1	1
MAS-CAU-AP-4	17.206 ± 13.924	27.587 ± 15.582	23.608 ± 14.726	25.294 ± 8.697	0.817	0.488	0.876	1	1	1	1	1
MAS-CAU-AP-5	9.075 ± 13.005	20.537 ± 16.086	15.91 ± 11.704	17.616 ± 7.322	1.449	0.234	0.371	0.789	0.989	1	1	1

MAS-CAU-AP-6	8.574 ± 12.417	14.37 ± 17.377	10.725 ± 12.598	11.501 ± 5.99	1.115	0.347	0.8	1	0.913	1	1	1
MAS-CAU-AP-7	-4.457 ± 11.339	-2.548 ± 12.315	-1.83 ± 14.546	-3.783 ± 8.153	0.2	0.896	1	1	1	1	1	1
LAS-CAU-AP-1	29.833 ± 20.253	26.038 ± 17.072	12.682 ± 21.155	25.4 ± 13.036	2.669	0.052	1	0.069	1	0.397	1	0.905
LAS-CAU-AP-2	46.807 ± 19.931	46.241 ± 25.181	34.905 ± 22	49.105 ± 14.149	1.521	0.214	1	0.377	1	1	1	1
LAS-CAU-AP-3	40.017 ± 17.227	38.971 ± 26.473	28.818 ± 18.297	36.609 ± 13.271	1.323	0.272	1	0.314	1	1	1	1
LAS-CAU-AP-4	29.892 ± 15.547	31.946 ± 25.865	20.114 ± 14.5	22.319 ± 12.631	2.227	0.090	1	0.121	0.412	1	1	1
LAS-CAU-AP-5	19.654 ± 14.39	28.158 ± 24.671	13.051 ± 12.922	14.3 ± 5.758	2.159	0.098	1	0.569	1	0.188	0.436	1
LAS-CAU-AP-6	10.633 ± 12.883	17.864 ± 14.674	6.732 ± 13.818	8.674 ± 6.36	1.294	0.281	1	1	1	0.322	1	1
LAS-CAU-AP-7	5.517 ± 12.232	3.016 ± 11.691	-5.252 ± 11.794	-1.585 ± 4.395	2.208	0.092	1	0.161	1	0.273	1	1
MAS-SN-VD-1	121.12 ± 52.793	158.536 ± 53.315	131.02 ± 67.218	197.712 ± 33.725	2.244	0.088	1	1	0.157	1	1	0.123
MAS-SN-VD-2	124.54 ± 48.481	163.855 ± 43.79	139.973 ± 51.444	182.41 ± 19.764	1.173	0.324	1	1	0.78	1	1	0.683
MAS-SN-VD-3	113.35 ± 36.805	153.739 ± 50.689	134.36 ± 41.44	154.25 ± 16.994	0.793	0.501	0.963	1	1	1	1	1
MAS-SN-VD-4	92.677 ± 34.293	125.949 ± 50.068	120.502 ± 43.077	123.144 ± 18.957	0.911	0.439	1	0.808	1	1	1	1
MAS-SN-VD-5	69.347 ± 32.794	89.632 ± 40.327	95.07 ± 45.895	84.504 ± 18.771	1.135	0.339	1	0.528	1	1	1	1
MAS-SN-VD-6	45.861 ± 34.382	61.55 ± 38.57	67.793 ± 46.717	45.803 ± 16.767	1.414	0.244	1	1	1	1	1	0.439
MAS-SN-VD-7	17.724 ± 38.699	37 ± 34.703	36.724 ± 47.151	9.543 ± 18.236	1.477	0.226	1	1	1	1	0.93	0.338
LAS-SN-VD-1	112.347 ± 50.251	142.484 ± 61.867	122.026 ± 75.292	137.961 ± 21.241	0.412	0.745	1	1	1	1	1	1
LAS-SN-VD-2	120.345 ± 45.873	155.614 ± 46.278	133.919 ± 48.524	159.34 ± 17.255	1.058	0.371	0.97	1	0.845	1	1	1
LAS-SN-VD-3	113.265 ± 37.744	148.924 ± 54.587	129.975 ± 44.4	158.542 ± 30.418	1.256	0.294	0.908	1	0.512	1	1	1
LAS-SN-VD-4	99.28 ± 33.751	124.609 ± 57.765	116.412 ± 46.388	137.804 ± 33.191	0.532	0.661	1	1	1	1	1	1
LAS-SN-VD-5	82.917 ± 34.298	94.804 ± 56.877	94.778 ± 45.827	104.202 ± 31.078	0.193	0.901	1	1	1	1	1	1
LAS-SN-VD-6	59.496 ± 40.92	69.232 ± 56.897	72.529 ± 50.39	74.49 ± 34.073	0.355	0.786	1	1	1	1	1	1
LAS-SN-VD-7	28.454 ± 47.848	37.826 ± 64.568	40.038 ± 54.497	46.316 ± 33.628	0.437	0.727	1	1	1	1	1	1
MAS-PUT-VD-1	-13.717 ± 14.98	-3.267 ± 24.083	-19.264 ± 17.338	-18.79 ± 11.038	4.096	**0.009**	**0.012**	1	1	**0.015**	0.824	1
MAS-PUT-VD-2	45.487 ± 24.175	69.656 ± 29.929	48.076 ± 26.32	59.376 ± 25.274	2.76	**0.047**	0.056	1	0.531	0.283	1	1
MAS-PUT-VD-3	51.277 ± 23.486	72.304 ± 24.197	55.843 ± 23.44	68.613 ± 19.257	1.738	0.165	0.294	1	0.621	1	1	1
MAS-PUT-VD-4	44.14 ± 21.52	60.55 ± 19.932	48.376 ± 25.156	56.976 ± 14.51	0.588	0.624	1	1	1	1	1	1
MAS-PUT-VD-5	33.331 ± 19.613	44.207 ± 19.75	36.03 ± 26.389	40.257 ± 13.753	0.085	0.968	1	1	1	1	1	1
MAS-PUT-VD-6	14.195 ± 17.268	16.916 ± 16.471	13.286 ± 22.041	13.01 ± 8.71	0.074	0.974	1	1	1	1	1	1
MAS-PUT-VD-7	-9.583 ± 11.974	-6.084 ± 11.949	-6.666 ± 13.169	-7.668 ± 6.566	0.536	0.659	1	1	1	1	1	1
LAS-PUT-VD-1	-9.204 ± 19.963	-0.156 ± 22.678	-16.021 ± 18.328	-11.551 ± 13.444	2.71	**0.050**	0.137	1	1	0.049	1	1
LAS-PUT-VD-2	44.016 ± 24.959	62.022 ± 25.305	36.782 ± 25.469	44.301 ± 27.825	2.362	0.076	0.253	1	1	0.083	1	1
LAS-PUT-VD-3	55.719 ± 25.949	70.948 ± 25.31	49.428 ± 19.512	64.862 ± 21.067	1.651	0.183	0.983	1	1	0.409	1	0.676
LAS-PUT-VD-4	51.079 ± 24.434	62.268 ± 26.924	46.536 ± 20.452	66.914 ± 17.47	1.08	0.362	1	1	1	1	1	0.568
LAS-PUT-VD-5	41.249 ± 24.474	47.997 ± 28.746	37.103 ± 22.832	55.609 ± 18.935	0.499	0.684	1	1	1	1	1	1
LAS-PUT-VD-6	20.285 ± 22.058	18.226 ± 26.157	18.93 ± 21.943	32.521 ± 19.298	0.648	0.586	1	1	1	1	1	1
LAS-PUT-VD-7	-1.253 ±	-8.124 ±	-4.271 ±	3.052 ±	0.977	0.407	0.831	1	1	1	1	1

	16.304	15.2	15.817	13.214								
MAS-CAU-VD-1	-10.352 ± 13.718	-15.406 ± 17.577	-11.395 ± 13.704	-19.542 ± 7.88	0.78	0.508	1	1	1	1	1	1
MAS-CAU-VD-2	-11.574 ± 15.152	-1.928 ± 19.506	-4.185 ± 21.24	-4.704 ± 12.389	0.724	0.540	0.901	1	1	1	1	1
MAS-CAU-VD-3	3.111 ± 14.953	11.661 ± 21.398	6.062 ± 22.908	19.605 ± 18.546	1.298	0.280	0.843	1	0.655	1	1	1
MAS-CAU-VD-4	29.661 ± 18.618	33.377 ± 23.805	25.855 ± 24.846	37.759 ± 18.277	0.578	0.631	1	1	1	1	1	1
MAS-CAU-VD-5	35.1 ± 14.769	40.443 ± 20.068	33.564 ± 18.443	42.665 ± 10.123	0.492	0.689	1	1	1	1	1	1
MAS-CAU-VD-6	23.14 ± 14.594	30.995 ± 16.268	28.106 ± 16.882	34.881 ± 10.214	0.741	0.530	1	1	1	1	1	1
MAS-CAU-VD-7	1.056 ± 11.003	7.242 ± 14.823	8.413 ± 13.757	8.18 ± 6.665	1.581	0.199	0.909	0.227	1	1	1	1
LAS-CAU-VD-1	-7.615 ± 14.09	-9.113 ± 13.743	-13.214 ± 16.161	-5.368 ± 7.201	0.858	0.466	1	1	1	1	1	1
LAS-CAU-VD-2	7.346 ± 18.41	-1.554 ± 14.304	-5.671 ± 16.438	-6.726 ± 8.056	1.42	0.242	1	0.282	1	1	1	1
LAS-CAU-VD-3	18.698 ± 15.082	14.901 ± 19.557	2.033 ± 16.207	9.371 ± 12.466	1.975	0.123	1	0.466	1	0.244	1	1
LAS-CAU-VD-4	40.76 ± 22.424	42.483 ± 23.731	24.669 ± 23.92	38.141 ± 16.145	1.956	0.126	1	0.435	1	0.279	1	0.969
LAS-CAU-VD-5	43.946 ± 19.516	46.33 ± 20.737	33.368 ± 19.083	45.61 ± 9.352	1.75	0.162	1	0.305	1	0.555	1	1
LAS-CAU-VD-6	37.787 ± 16.977	38.651 ± 24.825	26.31 ± 16.511	34.712 ± 11.504	1.988	0.121	1	0.132	1	0.673	1	1
LAS-CAU-VD-7	16.947 ± 12.661	11.549 ± 21.204	5.256 ± 15.107	10.849 ± 12.132	2.005	0.119	1	0.098	1	1	1	1
MAS-SN-ML-1	56.537 ± 42.048	88.062 ± 64.883	65.933 ± 51.691	78.23 ± 28.717	0.495	0.687	1	1	1	1	1	1
MAS-SN-ML-2	77.029 ± 41.1	101.244 ± 68.095	88.018 ± 53.178	97.023 ± 31.955	0.196	0.899	1	1	1	1	1	1
MAS-SN-ML-3	82.521 ± 37.486	115.501 ± 53.327	108.389 ± 52.935	113.291 ± 25.744	0.957	0.417	1	0.713	1	1	1	1
MAS-SN-ML-4	89.856 ± 36.386	121.61 ± 45.398	116.215 ± 44.242	125.618 ± 19.058	0.804	0.495	1	0.944	1	1	1	1
MAS-SN-ML-5	91.712 ± 38.169	120.107 ± 45.304	116.392 ± 43.472	125.957 ± 14.063	0.401	0.753	1	1	1	1	1	1
MAS-SN-ML-6	100.323 ± 45.898	129.296 ± 51.051	119.302 ± 50.906	133.149 ± 15.639	0.161	0.922	1	1	1	1	1	1
MAS-SN-ML-7	102.279 ± 53.193	137.768 ± 62.346	120.784 ± 59.528	134.278 ± 25.576	0.415	0.743	1	1	1	1	1	1
LAS-SN-ML-1	48.147 ± 32.739	67.018 ± 44.481	75.022 ± 49.102	56.626 ± 23.173	1.693	0.174	1	0.234	1	1	1	1
LAS-SN-ML-2	66.191 ± 37.363	92.94 ± 65.154	101.326 ± 54.23	78.442 ± 19.921	1.939	0.129	1	0.199	1	1	1	0.874
LAS-SN-ML-3	84.283 ± 34.63	110.577 ± 62.185	112.456 ± 51.309	104.217 ± 21.623	1.011	0.392	1	0.538	1	1	1	1
LAS-SN-ML-4	96.878 ± 35.064	122.138 ± 52.499	114.589 ± 45.225	128.925 ± 26.33	0.498	0.685	1	1	1	1	1	1
LAS-SN-ML-5	98.395 ± 37.706	121.679 ± 45.223	109.525 ± 43.603	138.327 ± 28.876	0.85	0.470	1	1	0.768	1	1	1
LAS-SN-ML-6	108.044 ± 45.991	132.124 ± 43.451	107.679 ± 48.55	152.338 ± 29.021	1.439	0.237	1	1	0.801	1	1	0.369
LAS-SN-ML-7	119.097 ± 55.545	140.353 ± 53.923	104.577 ± 54.884	156.426 ± 28.053	1.827	0.148	1	1	1	0.494	1	0.323
MAS-PUT-ML-1	-13.392 ± 13.923	-8.373 ± 22.426	-9.977 ± 20.217	-12.993 ± 8.786	0.36	0.782	1	1	1	1	1	1
MAS-PUT-ML-2	10.574 ± 18.815	20.072 ± 19.781	13.962 ± 25.752	12.496 ± 11.987	0.407	0.748	1	1	1	1	1	1
MAS-PUT-ML-3	33.066 ± 20.974	46.378 ± 21.625	36.815 ± 25.723	40.527 ± 13.864	0.641	0.590	1	1	1	1	1	1
MAS-PUT-ML-4	50.765 ± 23.65	65.862 ± 19.841	53.37 ± 23.889	62.709 ± 16.413	0.544	0.654	1	1	1	1	1	1
MAS-PUT-ML-5	46.92 ± 21.787	58.003 ± 20.748	44.503 ± 20.979	56.968 ± 15.192	0.535	0.659	1	1	1	1	1	1
MAS-PUT-ML-6	38.703 ± 20.578	60.978 ± 19.112	42.833 ± 16.709	56.571 ± 12.986	2.004	0.119	0.22	1	1	0.236	1	1
MAS-PUT-ML-7	58.719 ± 26.736	105.541 ± 34.405	68.446 ± 30.759	100.071 ± 26.028	5.806	**0.001**	**0.005**	1	0.101	**0.009**	1	0.104
LAS-PUT-ML-1	-2.422 ± 23.808	-4.759 ± 24.81	-12.997 ± 19.466	1.455 ± 10.427	1.152	0.332	1	1	1	1	1	0.764

LAS-PUT-ML-2	21.652 ± 22.974	22.057 ± 25.671	7.41 ± 22.1	28.998 ± 14.076	1.638	0.186	1	1	1	1	1	0.274
LAS-PUT-ML-3	40.678 ± 22.866	47.772 ± 26.042	32.605 ± 20.845	53.42 ± 17.525	1.326	0.271	1	1	0.822	1	1	0.35
LAS-PUT-ML-4	53.408 ± 22.469	66.324 ± 24.049	53.676 ± 21.202	68.493 ± 17.828	0.605	0.613	1	1	1	1	1	1
LAS-PUT-ML-5	44.712 ± 22.063	57.156 ± 20.486	50.376 ± 21.709	57.297 ± 17.09	0.057	0.982	1	1	1	1	1	1
LAS-PUT-ML-6	44.517 ± 20.932	57.274 ± 18.251	46.318 ± 17.935	57.388 ± 14.809	0.437	0.727	1	1	1	1	1	1
LAS-PUT-ML-7	74.608 ± 37.609	94.988 ± 41.855	62.047 ± 26.734	87.365 ± 28.339	2.395	0.073	1	0.869	1	0.167	1	0.316
MAS-CAU-ML-1	-9.612 ± 14.601	-19.447 ± 18.046	-12.278 ± 14.983	-20.51 ± 11.046	1.254	0.295	1	1	1	0.421	1	1
MAS-CAU-ML-2	-13.747 ± 13.692	-5.621 ± 17.8	-5.739 ± 16.151	-7.043 ± 6.425	1.743	0.164	0.305	0.329	1	1	1	1
MAS-CAU-ML-3	3.673 ± 15.931	13.138 ± 16.14	11.205 ± 18.685	15.482 ± 6.506	2.18	0.096	0.214	0.379	0.285	1	1	1
MAS-CAU-ML-4	16.351 ± 15.267	23.84 ± 16.19	22.111 ± 15.446	28.442 ± 7.749	1.163	0.328	1	1	0.617	1	1	1
MAS-CAU-ML-5	26.756 ± 14.619	34.342 ± 18.393	29.347 ± 16.022	36.94 ± 10.323	0.536	0.659	1	1	1	1	1	1
MAS-CAU-ML-6	35.103 ± 14.947	41.063 ± 22.448	34.606 ± 22.157	44.75 ± 13.513	0.325	0.807	1	1	1	1	1	1
MAS-CAU-ML-7	27.052 ± 24.775	20.757 ± 32.348	20.34 ± 30.324	20.95 ± 15.694	0.718	0.544	1	1	1	1	1	1
LAS-CAU-ML-1	-14.324 ± 15.401	-15.084 ± 15.468	-17.982 ± 16.889	-10.762 ± 7.565	0.624	0.601	1	1	1	1	1	1
LAS-CAU-ML-2	5.492 ± 17.365	-5.681 ± 24.086	-10.671 ± 15.889	-3.335 ± 4.814	2.321	0.080	0.647	0.065	1	1	1	1
LAS-CAU-ML-3	25.13 ± 17.058	17.658 ± 21.11	5.962 ± 16.012	13.073 ± 7.198	4.204	**0.008**	1	**0.004**	0.52	0.428	1	1
LAS-CAU-ML-4	31.067 ± 14.622	29.334 ± 19.517	17.145 ± 14.561	24.344 ± 9.551	3.273	**0.025**	1	**0.020**	1	0.300	1	1
LAS-CAU-ML-5	38.336 ± 15.987	42.091 ± 22.638	27.06 ± 15.362	37.788 ± 10.71	2.693	0.051	1	0.085	1	0.190	1	1
LAS-CAU-ML-6	45.727 ± 23.32	50.738 ± 28.378	37.579 ± 23.323	51.91 ± 16.337	0.882	0.454	1	1	1	1	1	1
LAS-CAU-ML-7	29.949 ± 26.504	24.904 ± 31.357	27.197 ± 29.353	28.91 ± 22.289	0.032	0.992	1	1	1	1	1	1

P-values for statistically significant differences after Bonferroni correction are shown in bold; A = Anterior; CAU, caudate; D = Dorsal; HC, healthy controls; LAS, less affected side; L = Lateral; MAS, more affected side; M = Medial; TD, tremor dominant subtype; TD-E, early disease stage of TD; TD-M, middle disease stage of TD; TD-L, late disease stage of TD; P = Posterior; PUT, putamen; SN, Substantia nigra; V = Ventral.

Reference

1. Drori E, Berman S, Mezer AA. Mapping microstructural gradients of the human striatum in normal aging and Parkinson's disease. *Sci Adv*. 2022;8(28):eabm1971. doi:10.1126/sciadv.abm1971
